# Effect of Different Phospholipids on α-Secretase Activity in the Non-Amyloidogenic Pathway of Alzheimer’s Disease

**DOI:** 10.3390/ijms14035879

**Published:** 2013-03-13

**Authors:** Marcus O. W. Grimm, Viola J. Haupenthal, Tatjana L. Rothhaar, Valerie C. Zimmer, Sven Grösgen, Benjamin Hundsdörfer, Johannes Lehmann, Heike S. Grimm, Tobias Hartmann

**Affiliations:** 1 Experimental Neurology, Saarland University, Kirrberger Str. 1, 66421 Homburg/Saar, Germany; E-Mails: viola.haupenthal@uniklinikum-saarland.de (V.J.H.); tatjana.rothhaar@uks.eu (T.L.R.); valerie-zimmer@web.de (V.C.Z.); sven.groesgen@uks.eu (S.G.); benjamin.hundsdoerfer@uks.eu (B.H.); lehmannjohannes87@web.de (J.L.); heike.grimm@gmx.de (H.S.G.); tobias.hartmann@uniklinikum-saarland.de (T.H.); 2 Neurodegeneration and Neurobiology, Saarland University, Kirrberger Str. 1, 66421 Homburg/Saar, Germany; 3 Deutsches Institut für DemenzPrävention (DIDP), Saarland University, Kirrberger Str. 1, 66421 Homburg/Saar, Germany

**Keywords:** Alzheimer’s disease, α-secretase, ADAM10, lipids, phospholipids, chain length, saturation, headgroup

## Abstract

Alzheimer’s disease (AD) is characterized by extracellular accumulation of amyloid-β peptide (Aβ), generated by proteolytic processing of the amyloid precursor protein (APP) by β- and γ-secretase. Aβ generation is inhibited when the initial ectodomain shedding is caused by α-secretase, cleaving APP within the Aβ domain. Therefore, an increase in α-secretase activity is an attractive therapeutic target for AD treatment. APP and the APP-cleaving secretases are all transmembrane proteins, thus local membrane lipid composition is proposed to influence APP processing. Although several studies have focused on γ-secretase, the effect of the membrane lipid microenvironment on α-secretase is poorly understood. In the present study, we systematically investigated the effect of fatty acid (FA) acyl chain length (10:0, 12:0, 14:0, 16:0, 18:0, 20:0, 22:0, 24:0), membrane polar lipid headgroup (phosphatidylcholine, phosphatidylethanolamine, phosphatidylserine), saturation grade and the FA double-bond position on α-secretase activity. We found that α-secretase activity is significantly elevated in the presence of FAs with short chain length and in the presence of polyunsaturated FAs, whereas variations in the phospholipid headgroups, as well as the double-bond position, have little or no effect on α-secretase activity. Overall, our study shows that local lipid membrane composition can influence α-secretase activity and might have beneficial effects for AD.

## 1. Introduction

Alzheimer’s disease (AD) is the most common cause of dementia among neurodegenerative diseases in the industrialized nations and is characterized by a progressive memory loss and cognitive failure. One of the main pathological hallmarks of AD is the aggregation of a small peptide, called amyloid-β (Aβ), as senile plaques in the brains of affected individuals [1,2]. Aβ is generated by sequential proteolytic processing of the amyloid precursor protein (APP), a large type-I transmembrane protein [3]. In the amyloidogenic pathway, APP is first cleaved by the transmembrane aspartic protease β-secretase BACE1 [4,5], generating the *N*-terminus of Aβ followed by a second cleavage at the γ-cleavage site within the transmembrane domain of APP, releasing toxic Aβ peptides [6,7]. The γ-secretase has been identified as a protein complex, consisting of at least four transmembrane proteins, presenilin1 (PS1) or presenilin2 (PS2), nicastrin, anterior pharynx defective 1 (APH1a or APH1b) and presenilin enhancer 2 (PEN2) [8–11]. In an alternative non-amyloidogenic pathway, APP can be processed by α-secretases, cleaving APP within the Aβ domain, resulting in a non-toxic peptide [12]. As the α- and β-secretases are assumed to compete for APP as a substrate, an increase in α-secretase cleavage is discussed to be a therapeutic approach for AD [13]. The α-secretases have been identified as members of the ADAM family (a disintegrin and metalloproteinase) [14–17], type-I transmembrane proteins of the metzincin family, requiring a zinc ion for their proteolytic activity. As APP itself and the secretases involved in APP cleavage are all integral membrane proteins, lipid composition of cellular membranes is discussed as influencing the proteolytic processing of APP [18–21], either by directly affecting secretase activities, or by modulating the dynamics and accessibility of APP to the cleaving proteases [22–24]. Moreover, it has been shown that the flexible transmembrane domain of APP is able to bind cholesterol [25]. Several lipids, including cholesterol, sphingomyeline and gangliosides, have been shown to alter amyloidogenic APP processing [22,26–31]. Furthermore, accumulating evidences suggest that neuronal phospholipid composition is altered in AD [32–34] and a recent study addressed the influence of membrane phospholipids on amyloidogenic processing of APP [35]. However, the effect of different phospholipids on non-amyloidogenic APP processing is poorly investigated. Phospholipids belong to glycerol-based lipids, which are characterized by a glycerol-backbone. The sn-1 and sn-2 position of the glycerol-backbone are esterified to a fatty acid (FA), while the sn-3 position is esterified to a phosphate group, which in turn is esterified to a polar headgroup. Phospholipids can therefore vary in different attributes, e.g., FA acyl chain length, saturation grade, double-bond position and in the polar lipid headgroup, greatly influencing the physicochemical properties of a specific phospholipid. As phospholipids determine the local microenvironment of transmembrane proteins and influence e.g., membrane fluidity, which is important for lateral movement of transmembrane proteins within the phospholipid bilayer, we systematically investigated the effect of specific phospholipids on α-secretase activity in different *in vitro* systems and in living cells.

## 2. Results and Discussion

### 2.1. Effect of FA Carbon Chain Length on α-Secretase Activity

In order to evaluate whether FA carbon chain length affects non-amyloidogenic processing of APP, we analyzed saturated FA chains of increasing carbon chain length with phosphatidylcholine (PC) as constant headgroup (PC10:0, PC12:0, PC14:0, PC16:0, PC18:0, PC20:0, PC22:0, PC24:0). In a first step, we prepared purified membranes of the human neuroblastoma cell line SH-SY5Y containing the membrane protein secretases involved in APP processing, incubated them with the PC phospholipids mentioned above, and measured α-secretase activity directly by *in vitro* processing of a fluorogenic α-secretase substrate. Uptake of the phospholipids was controlled by mass spectrometry (Figure S5).

**Figure 1:**
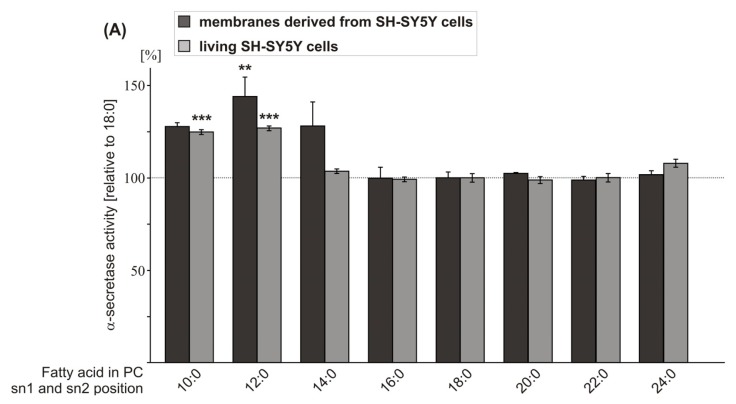

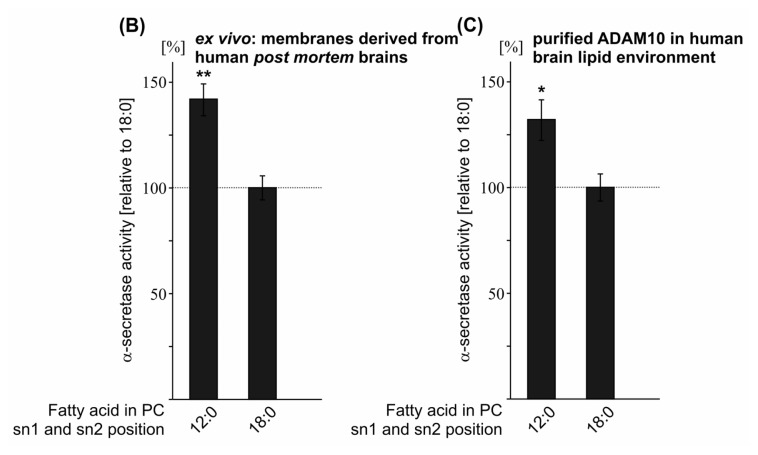
Effect of FA carbon chain length on α-secretase activity. (**A**) Purified membranes of SH-SY5Y wildtype (wt) cells and living SH-SY5Y wt cells were exposed to phospholipids containing FAs of increasing carbon chain length. For purified membranes, 25 μM phospholipid was used; living cells were exposed 8 + 16 h to 10 μM phospholipid. The α-secretase activity was measured by adding a fluorogenic α-secretase substrate and fluorescence was measured using a Safire^2^ Fluorometer; (**B**) Purified membranes of human *post mortem* brains were incubated with 50 μM PC12:0 or PC18:0. The α-secretase activity was determined as described for A; (**C**) Purified α-secretase ADAM10 was incubated with 25 μM PC12:0 or PC18:0 in the presence of human brain lipid extract. The α-secretase activity was determined as described for A. (**A**,**B**,**C**) All quantified data represent an average of at least three independent experiments. Error bars represent standard deviation of the mean. Asterisks show the statistical significance (*p ≤ 0.05; **p ≤ 0.01 and ***p ≤ 0.001).

As PC18:0 revealed no effect on α-secretase activity compared to purified SH-SY5Y membranes incubated with the solvent ethanol (Figure S1), and PC18:0 is one of the major PC species in the membrane (Table S5), PC18:0 represents the control FA throughout our study addressing FA carbon chain length. PC10:0, PC12:0 and PC14:0 increased α-secretase activity in purified membranes of SH-SY5Y wildtype (wt) cells (Figure 1A). However, statistical significance was only obtained for PC12:0 (PC10:0: 127.7% ± 2.2%, *p* = 0.123; PC12:0: 144.0% ± 9.3%, *p* = 0.002; PC14:0: 128.0% ± 11.9%, *p* = 0.116), whereas PC16:0, PC20:0, PC22:0 and PC24:0 showed no effect (Figure 1A, Table S1), thereby indicating that PC10:0, PC12:0 and PC14:0 increase α-secretase activity. Additionally, we evaluated the effect of increasing FA chain length on α-secretase activity in context of living cells. SH-SY5Y wt cells were cultured for 8 + 16 h in the presence of the phospholipids mentioned earlier (final concentration 10 μM) and α-secretase activity was measured by adding 10 μM phospholipid and 10 μM fluorogenic α-secretase substrate to the 96-well cell culture plate. In line with the efficient uptake of the phospholipids in purified membranes, the phospholipids were significantly increased after incubation in living SH-SY5Y cells. Uptake controls were shown for PC12:0 and PC18:0 (Figure S5). As already observed for purified SH-SY5Y wt membranes, PC10:0 and PC12:0 increased α-secretase activity (PC10:0: 124.8% ± 1.3%; *p* < 0.001; PC12:0: 126.9 ± 1.3%, *p* < 0.001) and PC16:0, PC20:0, PC22:0 and PC24:0 had no significant effect (Figure 1A, Table S1). However, as a slight difference compared to the results obtained from purified membranes, PC14:0 did not increase α-secretase activity in living cells (Figure 1A, Table S1). The discrepancy of the effect on α-secretase activity obtained for PC14:0 in purified membranes and living cells might be caused by additional factors present in a living system, e.g., altered gene expression or protein stability of α-secretase in the presence of PC14:0. Combining the α-secretase measurements of neuroblastoma SH-SY5Y wt cells, including purified membranes and living cells, revealed significance for PC10:0, PC12:0 and PC14:0 (combined data: PC10:0: 126.0% ± 1.3%, *p* < 0.001; PC12:0: 129.9% ± 2.4%, *p* < 0.001; PC14:0: 114.0 ± 6.7, *p* = 0.001) (Table S1), indicating that FA acyl chains with short chain length increase non-amyloidogenic processing of APP. Interestingly, for γ-secretase, it has been recently reported that increasing the FA carbon chain length (14, 16, 18 and 20) elevates γ-secretase activity [35], peaking at lengths of 18 and 20 carbons. The diverse effects of FAs with increasing carbon chain length on α- and γ-secretase activity might be caused by the presence of membrane microdomains with defined lipid and protein compositions. Detergent-resistant membrane microdomains, also called lipid rafts [36], containing higher levels of cholesterol and sphingolipids, are implicated in amyloidogenic APP processing [37]. The β-secretase BACE1 and amyloidogenic γ-secretase cleavage have been shown to be associated with lipid rafts [38–40]. Remarkably, lipid rafts also contain higher levels of FAs with 20 and 22 carbons [41]. In contrast to the involvement of lipid rafts in β- and γ-secretase processing of APP, the α-secretase mediated non-amyloidogenic processing is discussed to occur in non-raft regions of the membrane [42–44], which could be a possible explanation why phospholipids with longer FA carbon chains affect γ- but not α-secretase activity.

As phospholipid PC12:0 showed the strongest effect on α-secretase activity in purified membranes of SH-SY5Y wt cells and in living cells, we analyzed whether PC12:0 also increases non-amyloidogenic APP processing in the lipid environment of human brain. Therefore, purified membranes of nine healthy human *post mortem* brains were prepared and pooled, and α-secretase activity was measured in the presence of PC12:0. Indeed, PC12:0 also significantly elevated α-secretase activity in purified membranes of human *post mortem* brains compared to the control PC18:0 (PC12:0: 141.9% ± 7.6%, *p* = 0.004) (Figure 1B; Table S1). The increase was nearly identical as observed for purified SH-SY5Y wt membranes (PC12:0: 144.0% ± 9.3%, *p* = 0.002) (Figure 1A). Because these results suggest, that PC12:0 directly increases α-secretase activity, we further analyzed the effect of PC12:0 on purified enzyme. As the α-secretase ADAM10 has been recently shown to be the physiologically relevant α-secretase in neurons [45], we selected ADAM10 to determine the effect of phospholipids on purified α-secretase throughout this study. Purified ADAM10 was incubated with PC12:0 in the presence of human *post mortem* brain lipid extract to reconstitute brain lipid environment. Also in this experimental approach, ADAM10 activity was significantly increased in the presence of PC12:0 compared to PC18:0 (PC12:0: 132.0% ± 9.6%; *p* = 0.011) (Figure 1C, Table S1), validating that PC12:0 directly influences α-secretase activity. However, although ADAM10 is discussed to be the major α-secretase in neurons [45], further α-secretase candidates, e.g., ADAM17 and ADAM9 [14,16], might be affected by PC12:0. This is a rather likely scenario, as we obtained for purified membranes of SH-SY5Y wt cells and purified membranes of human *post mortem* brains, where total α-secretase and not exclusively ADAM10 activity is determined, an even stronger increase in α-secretase activity. However, our data suggest that ADAM10 is the α-secretase candidate mostly affected by shorter FA acyl chain length.

### 2.2. Variations in the Phospholipid Headgroup on α-Secretase Activity

**Figure 2:**
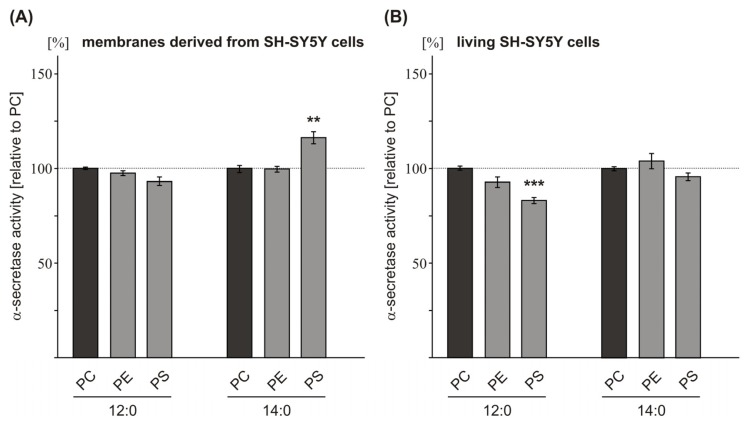

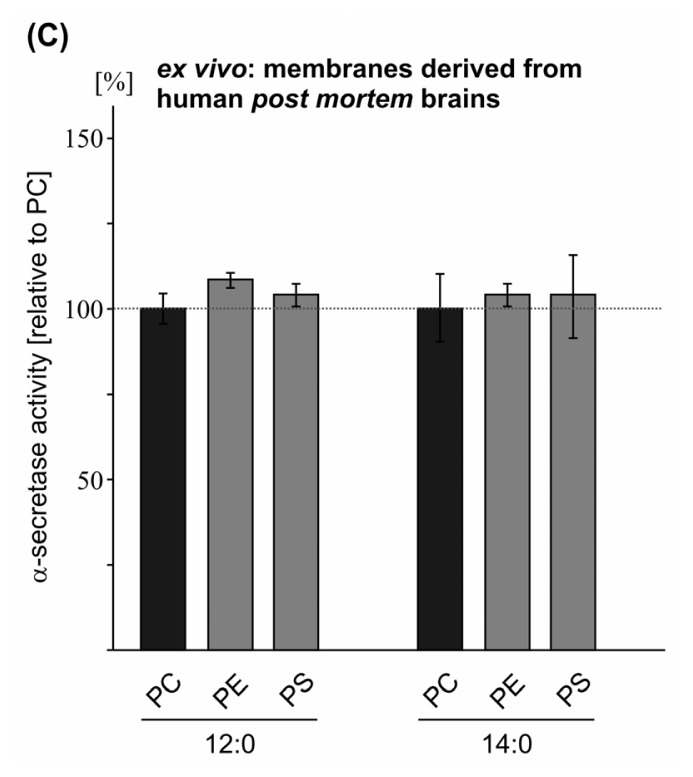
Effect of the phospholipid headgroup on α-secretase activity. Phospholipids having a constant FA chain length (12:0 and 14:0) but variable headgroups (PC, PE, PS) were incubated on (**A**) purified membranes of SH-SY5Y wt cells (**B**) living SH-SY5Y wt cells (**C**) purified membranes of human *post mortem* brains. (**A**,**B**,**C**) Phospholipid concentration, determination of α-secretase activity, illustration and statistical significance as described for Figure 1.

Beside FA carbon chain length the polar lipid headgroups of membrane phospholipids might affect non-amyloidogenic APP processing. As we observed increased α-secretase activity in the presence of PC10:0, PC12:0 and PC14:0, we examined whether changing the polar lipid headgroup to phosphatidylethanolamine (PE) or phosphatidylserine (PS) might additionally modulate the observed effect of short FA acyl chain length on α-secretase activity. In accordance with the analysis of the FA carbon chain length, we first measured α-secretase activity in purified membranes of SH-SY5Y wt cells exposed to different phospholipids, having a constant FA chain length (12:0 or 14:0) but variable headgroups (PC, PE, PS). No significant alterations in α-secretase activity were observed for PE12:0 and PE14:0 compared to PC12:0 or PC14:0 as control (Figure 2A, Table S2), indicating that a change in the phospholipid headgroup from PC to PE does not affect non-amyloidogenic α-secretase cleavage of APP. In contrast, changing PC to PS revealed some influence on α-secretase activity. However, there was no consistent effect on α-secretase activity in all experimental approaches as that observed for the FA acyl chain length. In detail, for PS14:0, we found a slight but nonetheless significant, increase in α-secretase activity in purified SH-SY5Y wt membranes, as compared to PC14:0 (PS14:0: 116.2% ± 3.1%, *p* = 0.001) (Figure 2A, Table S2). On the other hand, for PS12:0 no effect was observed, thereby indicating that the altered α-secretase activity found for PS14:0 is not only mediated by the variation in the phospholipid headgroup from PC to PS, but is also dependent on the FA chain length esterified to the PS phospholipid. Therefore we decided to calculate the effects on α-secretase activity in purified SH-SY5Y wt membranes observed for variable polar lipid headgroups independent of FA chain length. Indeed statistical analysis revealed no significant alterations on α-secretase activity for PE and PS compared to PC (PE: 99.7% +/− 1.0%, *p* = 1.000; PS: 102.0% ± 4.5%, *p* = 0.936) (Table S2), suggesting that a change in the polar headgroup alone does not affect α-secretase activity.

To further elucidate this, we analyzed the influence of variable phospholipid headgroups on α-secretase activity in living SH-SY5Y wt cells. In accordance to the results obtained with purified SH-SY5Y wt membranes, changing the phospholipid headgroup from PC to PE had no significant effect on α-secretase activity (Figure 2B, Table S2). In contrast to the results obtained with purified membranes of SH-SY5Y wt cells, PS12:0 significantly decreased α-secretase activity in living cells (PS12:0: 83.8% ± 1.6%, *p* = 0.003), whereas PS14:0 showed no significant effect compared to the corresponding PC phospholipids (Figure 2B, Table S2). Although a change in the polar lipid headgroup from PC to PS also seems to affect α-secretase cleavage of APP in living cells, at least for PS12:0, one has to take into consideration that, compared to the results obtained with purified membranes of SH-SY5Y wt cells, a significant effect was observed for chain length 12:0 and not for chain length 14:0 and α-secretase activity was reduced and not elevated. For that reason, from our results we cannot conclude whether a change from PC to PS *per se* in fact influences α-secretase activity and further experiments have to be done to address this topic. However, statistical analysis of the α-secretase measurements obtained for PE and PS independent of FA chain length in living cells again revealed no significant alterations on α-secretase activity (Table S2), suggesting that variations in the polar lipid headgroup independent of FA chain length might not affect α-secretase activity. Our hypothesis that a change in the phospholipid headgroup has no or only minor effects on α-secretase activity was also supported when purified membranes of human *post mortem* brains were exposed to the aforementioned phospholipids. Neither a change from PC to PE nor a change to PS in the phospholipid headgroup significantly affected α-secretase activity (Figure 2C, Table S2). The lack of an α-secretase effect when human brain membranes instead of SH-SY5Y wt membranes were incubated with PS14:0 might be explained by analyzing the PS to PC ratio in both membranes. TLC analysis of purified membranes (SH-SY5Y wt cells and human *post mortem* brains) revealed that the PS:PC ratio is lower in SH-SY5Y wt membranes compared to human brain membranes (Figure S2). Therefore, changing the phospholipid headgroup from PC to PS should have a more pronounced effect in SH-SY5Y wt cells compared to human brain membranes, as we were able to observe. This result indicates that the influence of a single phospholipid on the activity of membrane-imbedded enzymes strongly depends on the local lipid microenvironment. As Aβ deposition is a crucial event in AD and α-seretase cleavage prevents the formation of Aβ by forming a non-toxic peptide [12], we conclude from our results that changes in the phospholipid headgroup of phospholipid species with short FA acyl chain length have no significant effects on α-secretase activity in the lipid environment of human brain. Nevertheless, our results do not rule out that variations in the phospholipid headgroup might play a role in AD by affecting other molecular pathways besides α-secretase activity. Several studies addressed levels of the major membrane phospholipids in AD *post mortem* brains. PE and phosphatidylinositol (PI) levels have been reported to be significantly decreased in AD brains [33,34,46,47], whereas PS phospholipids are increased [33]. For PC Nitsch *et al.* reported a significant decrease in AD brains [47], whereas PC was unaffected in the study by Wells *et al.*[33]. In addition, mixtures of different phospholipid headgroup types, including PC, PE, PS, PI and phosphatidic acid (PA) have been shown to alter γ-secretase cleavage *in vitro*[19,35]. Furthermore, recently Nesic *et al.* reported, that α-secretase non-amyloidogenic APP processing is increased when PE synthesis was inhibited in mammalian HEK293 cells whereas γ-secretase cleavage of APP is reduced, resulting in decreased Aβ levels [48].

### 2.3. Effect of FA Saturation on α-Secretase Activity

**Figure 3:**
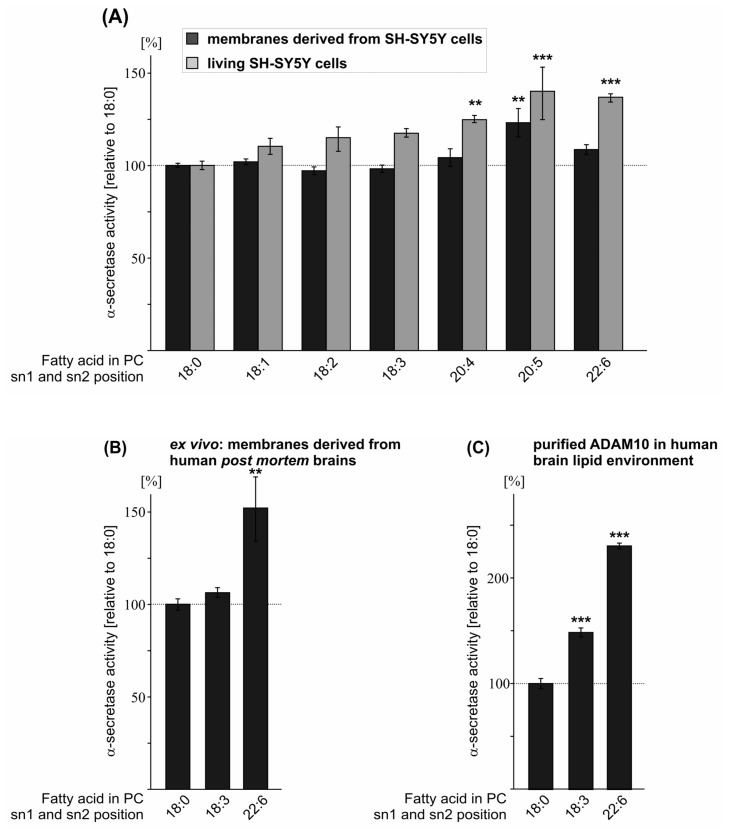
Effect of FA saturation on α-secretase activity. Phospholipids containing monoor polyunsaturated FAs were exposed to (**A**) purified membranes of SH-SY5Y wt cells and living SH-SY5Y wt cells (**B**) purified membranes of human *post mortem* brains (**C**) purified α-secretase ADAM10 in the presence of human brain lipid extract. (**A**,**B**,**C**) Phospholipid concentration, determination of α-secretase activity, illustration and statistical significance as described for Figure 1.

Another important feature of membrane phospholipids is the saturation grade of the FA acyl chains. In addition to FA chain length, the FA saturation grade strongly affects membrane fluidity, which is important for membrane-dependent cellular functions, e.g., cell signaling, transmembrane protein function, lateral diffusion of membrane proteins within the membrane, protein–lipid interactions and vesicle formation [49]. To investigate whether FA acyl chain saturation of phospholipids influences the proteolytic activity of α-secretase, we analyzed monounsaturated and polyunsaturated FA chains esterified to a PC glycerol-backbone compared to saturated stearic acid (PC18:0, PC18:1, PC18:2, PC18:3, PC20:4, PC20:5, PC22:6). Moreover, these FAs represent the most dominant species found in brain (Table S5). Treatment of purified SH-SY5Y wt membranes with oleic acid (PC18:1 Δ9-cis), linoleic acid (PC18:2), linolenic acid (PC18:3), arachidonic acid (AA, PC20:4) and docosahexaenoic acid (DHA, PC22:6) showed no significant changes in α-secretase activity compared to the control phospholipid PC18:0 (Figure 3A, Table S3). FA side chain eicosapentaenoic acid (EPA, PC20:5) significantly elevated α-secretase activity (EPA/PC20:5: 123.1% ± 7.6%, *p* = 0.005) (Figure 3A, Table S3). Exposing living SH-SY5Y wt cells to the aforementioned phospholipids (final concentration 10 μM) revealed significantly increased α-secretase activity in the presence of arachidonic acid, EPA and DHA, compared to control PC18:0 (AA/PC20:4: 124.7% ± 2.0%, *p* = 0.001; EPA/PC20:5: 140.1% ± 14.2%, *p* < 0.001; DHA/PC22:6: 136.8% ± 2.2%, *p* < 0.001) (Figure 3A, Table S3). Interestingly, in living cells, α-secretase activity was elevated with an increasing number of double bonds within the acyl chains with the strongest increase for EPA and DHA (Figure 3A), indicating that EPA and DHA are potent stimulators of non-amyloidogenic APP processing in a living system. Calculating all α-secretase measurements for SH-SY5Y wt cells, including purified membranes and living cells, showed similar results. Significance was obtained for arachidonic acid, EPA and DHA (AA/PC20:4: 117.9% ± 3.5%, *p* = 0.002; EPA/PC20:5: 132.5% ± 5.9%, *p* < 0.001; DHA/PC22:6: 123.5% ± 3.9%, *p* < 0.001) (Table S3).

To analyze whether polyunsaturated FAs also increase α-secretase activity in the lipid environment of human brain, we incubated stearic acid (18:0), linolenic acid (18:3) and DHA (22:6), which is highly enriched in neuronal membranes [50–52], on purified membranes of human *post mortem* brains. As already observed for purified SH-SY5Y wt membranes and for living cells, linolenic acid showed non-significant alterations on α-secretase activity and DHA again significantly elevated α-secretase activity (DHA: 152.0% ± 17.4, *p* = 0.007) (Figure 3C, Table S3). Using purified ADAM10 enzyme for the incubation with linolenic acid (PC18:3) and DHA (PC22:6) in the presence of human *post mortem* brain lipid extract, revealed that ADAM10 activity was significantly increased for both polyunsaturated FAs (PC18:3: 148.2% ± 4.2%, *p* < 0.001; DHA/PC22:6: 229.7% ± 2.7%, *p* < 0.001) (Figure 3C, Table S3). The extraordinary strong increase obtained with purified ADAM10 enzyme might be explained by the lack of other membrane proteins potentially involved in α-secretase regulation using brain lipid extract in contrast to purified membranes of human brains, which are not devoid of integral membrane proteins. One might speculate that such a protein could be e.g., β-secretase BACE1, which competes with α-secretase for the initial cleavage of the substrate APP. In purified membranes of human *post mortem* brains BACE1 is still present and can cleave the APP substrate resulting in lower α-secretase cleavage compared to the α-secretase measurements using purified α-secretase ADAM10 and brain lipid extract where BACE1 is lacking and ADAM10 has not to compete with BACE1 for APP cleavage. In summary, all our results indicate that FA acyl chain saturation of phospholipids affects α-secretase activity with a more pronounced effect with an increasing number of double bonds within the hydrocarbon chains of the FAs. Arachidonic acid (four double bonds), EPA (five double bonds) and DHA (six double bonds) significantly increased α-secretase activity in living SH-SY5Y wt cells, whereas FAs with less than four double bonds revealed minor or no significant effects. This finding is in line with the recent study by Yang *et al.*, showing increased levels of α-secreted APP (sAPPα) in the culture medium of differentiated SH-SY5Y cells exposed to arachidonic acid (20:4), EPA (20:5) and DHA (22:6) [53]. In the presence of DHA sAPPα secretion has also been shown to be increased in HEK cells expressing the substrate APP [54]. Additionally, stearic acid (18:0), oleic acid (18:1) and linoleic acid (18:2) showed no changes in sAPPα secretion in the study by Yang *et al.*[53], validating our results obtained by measuring α-secretase activity by the use of fluorogenic peptide. Furthermore, we validated our assay by using α-secretase inhibitors. Utilizing these inhibitors, α-secretase activity was decreased and for the non-cytotoxic α-secretase inhibitor GM6001, a reduction of sAPPα was also observed (Figure S4). However, it cannot be ruled out that the natural substrate, the intact APP protein, has different physical properties, e.g., affinity to the α-secretases, compared to the fluorogenic peptide and that these differences might influence the effect strength of phospholipids on α-secretase activity.

Among the FAs analyzed, the main focus has to concentrate on DHA. DHA is highly enriched in neuronal cell membranes [50–52] and DHA levels have been found to be decreased in the brain and serum of AD patients and in AD *post mortem* brains [55–57]. Furthermore, several epidemiological studies indicate an inverse correlation between DHA intake and AD incidence or cognitive decline [58–61]. Importantly, DHA has also been shown to reduce Aβ generation *in vitro* and in animal models of AD [35,62–68]. These findings, together with our finding that DHA strongly increases non-amyloidogenic α-secretase activity in the lipid environment of human brain, indicate that DHA might have protective effects on AD by decreasing amyloidogenic and increasing non-amyloidogenic APP processing.

### 2.4. Effect of the Double-Bond Position on α-Secretase Activity

**Figure 4:**
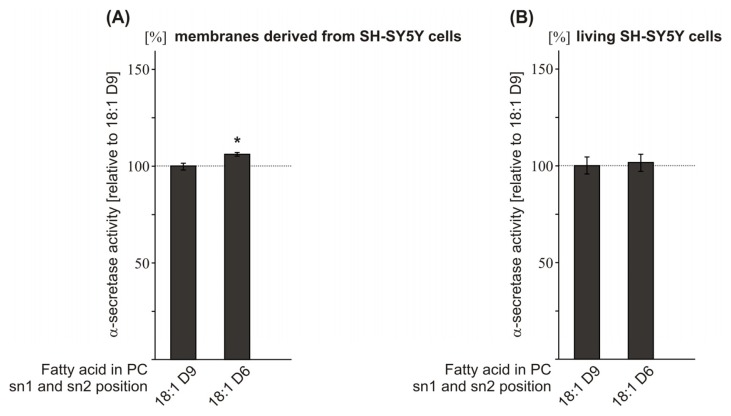
Effect of the double-bond position within the FA carbon chain on α-secretase activity. Phospholipids containing either FA 18:1Δ9-cis or FA 18:1Δ6-cis were incubated on (**A**) purified membranes of SH-SY5Y wt cells and (**B**) living SH-SY5Y wt cells; (**A**,**B**) Phospholipid concentration, determination of α-secretase activity, illustration and statistical significance as described for Figure 1.

As we observed that FA chain length and the saturation grade of FAs influence α-secretase activity, we asked whether shifting the position of the double-bond within the monounsaturated FA oleic acid PC18:1 might also affect α-secretase activity. Therefore, as a proof of principle, we analyzed PC18:1 Δ6-cis and PC18:1 Δ9-cis. Exposure of purified SH-SY5Y wt membranes to the Δ6-cis isomer revealed slightly—though significantly—increased α-secretase activity compared to PC18:1 Δ9-cis (PC18:1 Δ6-cis: 106.1% ± 1.0%, *p* = 0.041) (Figure 4A, Table S4), whereas no effect on α-secretase activity was obtained in living SH-SY5Y wt cells (Figure 4B, Table S4), thereby indicating that changing the position of the double-bond within a monounsaturated FA chain has no or only minor effects on non-amyloidogenic processing of APP. However, shifting the double-bond position further from the center of the membrane bilayer has been recently shown to affect γ-secretase cleavage [35]. In this study, γ-secretase activity was reduced in PC18:1Δ6-cis compared to PC18:1Δ9-cis. The observation that γ-secretase activity is affected more strongly by the position of the double-bond-induced kink within the phospholipid bilayer as α-secretase activity might be explained by the fact that γ-secretase cleaves the substrate APP within the middle of the phospholipid bilayer [69], whereas α-secretase cleaves APP within the extracellular/luminal domain.

## 3. Experimental Section

### 3.1. Chemicals and Reagents

All chemicals used here were purchased from Sigma Aldrich (Taufkirchen, Germany), if not stated otherwise. Complete protease inhibitor cocktail was purchased from Roche (Grenzach-Wyhlen, Germany), Aspartate protease inhibitor, β-secretase inhibitor, and fluorogenic substrate were purchased from Calbiochem (Darmstadt, Germany). The following phospholipids were purchased from Avanti Polar Lipids (Alabaster, AL, USA), except for PC20:5 (Sigma, Taufkirchen, Germany): **PC10:0**—1,2-didecanoyl-*sn*-glycero-3-phosphocholine; **PC12:0**—1,2-dilauroyl-*sn*-glycero-3-phosphocholine; **PC14:0**—1,2-dimyristoyl-*sn*-glycero-3-phosphocholine; **PC16:0**—1,2-dipalmitoyl*sn*-glycero-3-phosphocholine; **PC18:0**—1,2-distearoyl-*sn*-glycero-3-phosphocholine; **PC20:0**—1,2-diarachidoyl-*sn*-glycero-3-phosphocholine; **PC22:0**—1,2-dibehenoyl-*sn*-glycero-3-phosphocholine; **PC24:0**—1,2-dilignoceroyl-*sn*-glycero-3-phosphocholine; **PC18:1D9**—1,2-dioleoyl-*sn*-glycero-3-phosphocholine; **PC18:1D6**—1,2-dipetroselenoyl-*sn*-glycero-3-phosphocholine; **PC18:2**—1,2-dilinoleoyl-*sn*-glycero-3-phosphocholine; **PC18:3**—1,2-dilinolenoyl-*sn*-glycero-3-phosphocholine; **PC20:4—**1,2-diarachidonoyl-*sn*-glycero-3-phosphocholine; **PC20:5**—1,2-dieicosapentaenoyl-*sn*glycero-3-phosphocholine; **PC22:6**—1,2-didocosahexaenoyl-*sn*-glycero-3-phosphocholine; **PE12:0**1,2-dilauroyl-*sn*-glycero-3-phosphoethanolamine; **PE14:0**—1,2-dimyristoyl-*sn*-glycero-3-phosphoethanolamine; **PS12:0**—1,2-dilauroyl-*sn*-glycero-3-phospho-L-serine; **PS14:0**—1,2-dimyristoyl-*sn*-glycero-3-phospho-l-serine.

### 3.2. Cell Culture and Incubation with Phospholipids

SH-SY5Ywt cells were cultivated in Dulbecco’s Modified Eagle’s Medium supplemented with 10% FCS (PAN Biotech, Aidenbach, Germany) and non-essential amino acid solution. For incubation with phospholipids, SH-SY5Ywt cells were plated on a 96-well plate (Thermo Scientific, Waltham, MA, USA) and grown until confluency. Before incubation, cells were cultivated in medium containing 0.1% FCS for 4 h. Incubation was carried out for 8 h + 16 h with 10 μM phospholipid. Final concentration of ethanol was less than 2‰, except PE- and PS-species (10‰).

### 3.3. Detection of α-Secretase Activity *in Vivo*

After incubation, SH-SY5Ywt cells were washed once with HEPES buffer, then 100 μL of cell imaging solution (140 mM NaCl_2_, 5 mM KCl, 8 mM CaCl_2_, 1 mM MgCl_2_, 20 mM HEPES, pH 7.4) containing 10 μM phospholipid and 10 μM fluorogenic α-secretase substrate (sequence: Ac-RE-EDANS-VHHQK LVF-K-DABCYL-R-OH, Cat. No. 565767, Calbiochem, Darmstadt, Germany) was added. Fluorescence was measured continuously for 10,000 s with an excitation wavelength of 340 ± 10 nm and an emission wavelength of 490 ± 10 nm at 37 °C under light exclusion using a Safire^2^ Fluorometer (Tecan, Crailsheim, Germany) [23]. Enzyme activity kinetics are shown in the supplemental data section.

### 3.4. Human *Post Mortem* Brains

In total, nine human *post mortem* control brain samples were used. Brains were obtained from BrainNet (Munich, Germany). No CERAD status was assigned to 4 out of 5 brain samples. The fifth brain sample was assigned with CERAD status 0 (CERAD = the consortium to establish a registry for AD, standardizing procedures for the evaluation and diagnosis if patients with AD. A, B, C, 0 as described in http://cerad.mc.duke.edu/ [70]).

### 3.5. Preparation of Purified Membranes

SH-SY5Ywt cells and human *post mortem* brains were homogenized using a PotterS (Braun, Melsungen, Germany) at maximum speed (25 strokes) on ice. Protein concentration was measured according to Smith *et al.*[71], as described in detail earlier [72]. Samples were adjusted to 1 mg/mL, centrifuged at 900 rcf for 10 min at 4 °C and the obtained post-nuclear fractions were centrifuged at 55,000 rpm for 75 min at 4 °C. The pellet with purified membranes was resuspended using cannulaes (BD, Franklin Lakes, NJ, USA) with decreasing diameter in sucrose buffer (200 mM sucrose, 5 μM CaCl_2_, 5 μM ZnCl_2_) according to Grimm *et al.*[68].

### 3.6. Preparation of Human Brain Lipid Extract

Lipids were extracted according to Bligh and Dyer [73] with minor modifications [72,74]. In brief, 3.75 mL CHCl_3_:MeOH:HCl (37%) (1:2:0.06; *v*/*v*/*v*) was added to the sample and the mixture was vortexed for 1 h at room temperature (RT). Then, 1.25 mL CHCl_3_ was added and vortexed again for 1 h at RT. Finally, 1.25 mL CHCl_3_ and 1.25 mL H_2_O were added and samples were vortexed for another 10 min before centrifugation at 5000 rpm for 10 min. The lower phase containing lipids was transferred to another glass tube and evaporated under nitrogen flow at 30 °C. Lipids were resuspended in 1 mL H_2_O and the extraction cycle described earlier was repeated before lipids were finally dissolved in ethanol.

### 3.7. Determination of α-Secretase Activity and *in Vitro* Incubation

Post-nuclear fractions of SH-SY5Ywt cells/human brain-purified membranes were incubated with 25 μM/50 μM phospholipids for 15 min at 37 °C while shaking (Multireax, Heidolph Instruments, Schwabach, Germany). For determination of α-secretase activity, samples were adjusted to 100 μg of protein and 4 μM of fluorogenic α-secretase substrate was added. Fluorescence was measured as described above. Enzyme activity kinetics are shown in the supplemental data section.

### 3.8. Determination of ADAM10 Purified Enzyme Activity

Phospholipids (25 μM) were mixed with human brain lipid extract (5 μg lipid per 100 μL sucrose buffer) and incubated with 100 ng of ADAM10 purified enzyme (Cat. no. A9975, Sigma Aldrich, Taufkirchen, Germany) in sucrose buffer for 1 h at 4 °C while shaking, followed by sonification for 15 min on ice. ADAM10 purified enzyme activity was determined as described above using the Infinite M1000 Pro Fluorometer. Enzyme activity kinetics are shown in the supplemental data section.

### 3.9. Detection of Phospholipid Species in SH-SY5Ywt Cells and Human *Post Mortem* Brains

Lipid extraction was performed as described above. One hundred microliter of each sample were spotted on thin layer chromatography plate and lipids were analyzed as described before [75].

### 3.10. Mass Spectrometry Analysis

To determine phospholipid distribution in post-nuclear fractions and membranes derived from human brains, phosphatidylcholine amounts were measured as described and validated in detail earlier [76]. For analysis, we used 20 μL of post-nuclear fractions and human brain lipid extract (for preparations see sections before). According to this method, additionally, the phospholipid uptake into living cells and membranes of SH-SY5Y cells was determined.

### 3.11. Statistical Analysis

All quantified data presented here is based on an average of at least three independent experiments. Error bars represent standard deviation of the mean. Statistical significance was determined by ANOVA or two-tailed Student’s *t*-test; significance was set at *p ≤ 0.05; **p ≤ 0.01 and ***p ≤ 0.001.

## 4. Conclusions

Our results suggest that changes in the phospholipid composition of plasma membranes can influence α-secretase activity (Table 1) and might be involved in the development of AD. We found that α-secretase activity was significantly increased in the presence of FAs with short chain length and in the presence of polyunsaturated FAs with more than three double-bonds, thus suggesting that an increase in these phospholipids might have beneficial effects for AD.

## Supplementary Information



## Figures and Tables

**Table 1:** Summary of the effects of different phospholipids on α-secretase activity.

	SH-SY5Y membranes	living SH-SY5Y cells	purified ADAM10	human *post mortem* brain

	Mean % (SEM % +/− Sign.)	Mean % (SEM % +/− Sign.)	Mean % (SEM % +/− Sign.)	Mean % (SEM % +/− Sign.)
Effect of chain length				
PC 10:0	127.7 (2.2 n.s.)	124.8 (1.3 ***)		
PC 12:0	144.0 (9.3 **)	126.9 (1.3 ***)	132.0 (9.6 *)	141.9 (7.6 **)
PC 14:0	128.0 (11.9 n.s.)	103.5 (1.2 n.s.)		
PC 16:0	99.8 (5.9 n.s.)	99.2 (1.3 n.s.)		
PC 18:0	100.0 (3.2)	100.0 (2.3 n.s.)	100.0 (6.4)	100.0 (5.7)
PC 20:0	102.3 (0.5 n.s.)	98.8 (1.9 n.s.)		
PC 22:0	98.8 (1.9 n.s.)	100.1 (2.3 n.s.)		
PC 24:0	101.7 (1.5 n.s.)	107.8 (2.2 n.s.)		

	**Mean % (SEM % +/− Sign.)**	**Mean % (SEM % +/− Sign.)**	**Mean % (SEM % +/− Sign.)**	**Mean % (SEM % +/− Sign.)**
Effect of headgroup				

PC 12:0	100.0 (0.7)	100.0 (1.1)		100.0 (4.5)
PE 12:0	98.5 (1.3 n.s.)	93.6 (2.8 n.s.)		109.2 (3.1 n.s.)
PS 12:0	93.4 (2.5 n.s.)	83.8 (1.6 **)		104.9 (3.7 n.s.)
PC 14:0	100.0 (1.9)	100.0 (1.1)		100.0 (10.9)
PE 14:0	101.0 (1.5 n.s.)	105.0 (4.0 n.s.)		105.7 (3.6 n.s.)
PS 14:0	116.2 (3.1 ***)	96.6 (2.0 n.s.)		104.7 (12.2 n.s.)

	**Mean % (SEM % +/− Sign.)**	**Mean % (SEM % +/− Sign.)**	**Mean % (SEM % +/− Sign.)**	**Mean % (SEM % +/− Sign.)**
Effect of saturation				

PC 18:0	100.0 (1.2)	100.0 (2.3)	100.0 (4.8)	100.0 (3.1)
PC 18:1	102.0 (1.5 n.s.)	110.3 (4.3 n.s.)		
PC 18:2	97.1 (2.1 n.s.)	115.0 (6.6 n.s.)		
PC 18:3	98.2 (2.0 n.s.)	117.4 (2.3 n.s.)	148.2 (4.2 ***)	106.3 (2.7 n.s.)
PC 20:4	104.2 (4.8 n.s.)	124.7 (2.0 **)		
PC 20:5	123.1 (7.6 **)	140.1 (14.2 ***)		
PC 22:6	108.6 (2.6 n.s.)	136.8 (2.2 ***)	229.7 (2.7 ***)	152.0 (17.4 **)

	**Mean % (SEM % +/− Sign.)**	**Mean % (SEM % +/− Sign.)**	**Mean % (SEM % +/− Sign.)**	**Mean % (SEM % +/− Sign.)**
Effect of double-bond position				

PC 18:1D9	100.0 (1.8)	100.0 (4.4)		
PC 18:1D6	106.1 (1.0 *)	101.7 (4.5 n.s.)		

Asterisks show the statistical significance (

* *p* ≤ 0.05;

** *p* ≤ 0.01 and

*** *p* ≤ 0.001, n.s. not significant).

## References

[1] Masters C.L., Simms G., Weinman N.A., Multhaup G., McDonald B.L., Beyreuther K. (1985). Amyloid plaque core protein in Alzheimer disease and Down syndrome. Proc. Natl. Acad. Sci. USA.

[2] Selkoe D.J. (2001). Alzheimer’s disease: Genes, proteins, and therapy. Physiol. Rev.

[3] Dyrks T., Weidemann A., Multhaup G., Salbaum J.M., Lemaire H.G., Kang J., Muller-Hill B., Masters C.L., Beyreuther K. (1988). Identification, transmembrane orientation and biogenesis of the amyloid A4 precursor of Alzheimer’s disease. EMBO J.

[4] Vassar R., Bennett B.D., Babu-Khan S., Kahn S., Mendiaz E.A., Denis P., Teplow D.B., Ross S., Amarante P., Loeloff R. (1999). Beta-secretase cleavage of Alzheimer’s amyloid precursor protein by the transmembrane aspartic protease BACE. Science.

[5] Sinha S., Anderson J.P., Barbour R., Basi G.S., Caccavello R., Davis D., Doan M., Dovey H.F., Frigon N., Hong J. (1999). Purification and cloning of amyloid precursor protein beta-secretase from human brain. Nature.

[6] Haass C., Schlossmacher M.G., Hung A.Y., Vigo-Pelfrey C., Mellon A., Ostaszewski B.L., Lieberburg I., Koo E.H., Schenk D., Teplow D.B. (1992). Amyloid beta-peptide is produced by cultured cells during normal metabolism. Nature.

[7] Shoji M., Golde T.E., Ghiso J., Cheung T.T., Estus S., Shaffer L.M., Cai X.D., McKay D.M., Tintner R., Frangione B. (1992). Production of the Alzheimer amyloid beta protein by normal proteolytic processing. Science.

[8] Herreman A., Serneels L., Annaert W., Collen D., Schoonjans L., de Strooper B. (2000). Total inactivation of gamma-secretase activity in presenilin-deficient embryonic stem cells. Nat. Cell. Biol.

[9] Takasugi N., Tomita T., Hayashi I., Tsuruoka M., Niimura M., Takahashi Y., Thinakaran G., Iwatsubo T. (2003). The role of presenilin cofactors in the gamma-secretase complex. Nature.

[10] Kimberly W.T., LaVoie M.J., Ostaszewski B.L., Ye W., Wolfe M.S., Selkoe D.J. (2003). Gamma-secretase is a membrane protein complex comprised of presenilin, nicastrin, Aph-1, and Pen-2. Proc. Natl. Acad. Sci. USA.

[11] Duering M., Grimm M.O., Grimm H.S., Schroder J., Hartmann T. (2005). Mean age of onset in familial Alzheimer’s disease is determined by amyloid beta 42. Neurobiol. Aging.

[12] Haass C., Hung A.Y., Schlossmacher M.G., Teplow D.B., Selkoe D.J. (1993). Beta-Amyloid peptide and a 3-kDa fragment are derived by distinct cellular mechanisms. J. Biol. Chem.

[13] Lichtenthaler S.F. (2011). Alpha-secretase in Alzheimer’s disease: Molecular identity, regulation and therapeutic potential. J. Neurochem.

[14] Buxbaum J.D., Liu K.N., Luo Y., Slack J.L., Stocking K.L., Peschon J.J., Johnson R.S., Castner B.J., Cerretti D.P., Black R.A. (1998). Evidence that tumor necrosis factor alpha converting enzyme is involved in regulated alpha-secretase cleavage of the Alzheimer amyloid protein precursor. J. Biol. Chem.

[15] Lammich S., Kojro E., Postina R., Gilbert S., Pfeiffer R., Jasionowski M., Haass C., Fahrenholz F. (1999). Constitutive and regulated alpha-secretase cleavage of Alzheimer’s amyloid precursor protein by a disintegrin metalloprotease. Proc. Natl. Acad. Sci. USA.

[16] Koike H., Tomioka S., Sorimachi H., Saido T.C., Maruyama K., Okuyama A., Fujisawa-Sehara A., Ohno S., Suzuki K., Ishiura S. (1999). Membrane-anchored metalloprotease MDC9 has an alpha-secretase activity responsible for processing the amyloid precursor protein. Biochem. J.

[17] Allinson T.M., Parkin E.T., Turner A.J., Hooper N.M. (2003). ADAMs family members as amyloid precursor protein alpha-secretases. J. Neurosci. Res.

[18] Hartmann T., Kuchenbecker J., Grimm M.O. (2007). Alzheimer’s disease: The lipid connection. J. Neurochem.

[19] Osenkowski P., Ye W., Wang R., Wolfe M.S., Selkoe D.J. (2008). Direct and potent regulation of gamma-secretase by its lipid microenvironment. J. Biol. Chem.

[20] Grimm M.O., Rothhaar T.L., Hartmann T. (2012). The role of APP proteolytic processing in lipid metabolism. Exp. Brain Res.

[21] Lemkul J.A., Bevan D.R. (2011). Lipid composition influences the release of Alzheimer’s amyloid beta-peptide from membranes. Protein Sci.

[22] Grimm M.O., Rothhaar T.L., Grösgen S., Burg V.K., Hundsdorfer B., Haupenthal V.J., Friess P., Kins S., Grimm H.S., Hartmann T. (2012). Trans fatty acids enhance amyloidogenic processing of the Alzheimer amyloid precursor protein (APP). J. Nutr. Biochem.

[23] Rothhaar T.L., Grosgen S., Haupenthal V.J., Burg V.K., Hundsdorfer B., Mett J., Riemenschneider M., Grimm H.S., Hartmann T., Grimm M.O. (2012). Plasmalogens inhibit APP processing by directly affecting gamma-secretase activity in Alzheimer’s disease. Sci. World J.

[24] Marenchino M., Williamson P.T., Murri S., Zandomeneghi G., Wunderli-Allenspach H., Meier B.H., Kramer S.D. (2008). Dynamics and Cleavability at the alpha-cleavage site of APP(684–726) in different lipid environments. Biophys. J.

[25] Barrett P.J., Song Y., van Horn W.D., Hustedt E.J., Schafer J.M., Hadziselimovic A., Beel A.J., Sanders C.R. (2012). The amyloid precursor protein has a flexible transmembrane domain and binds cholesterol. Science.

[26] Simons M., Keller P., de Strooper B., Beyreuther K., Dotti C.G., Simons K. (1998). Cholesterol depletion inhibits the generation of beta-amyloid in hippocampal neurons. Proc. Natl. Acad. Sci. USA.

[27] Grösgen S., Grimm M.O., Friess P., Hartmann T. (2010). Role of amyloid beta in lipid homeostasis. Biochim. Biophys. Acta.

[28] Grimm M.O., Grimm H.S., Tomic I., Beyreuther K., Hartmann T., Bergmann C. (2008). Independent inhibition of Alzheimer disease beta- and gamma-secretase cleavage by lowered cholesterol levels. J. Biol. Chem.

[29] Grimm M.O., Grimm H.S., Patzold A.J., Zinser E.G., Halonen R., Duering M., Tschape J.A., De Strooper B., Muller U., Shen J. (2005). Regulation of cholesterol and sphingomyelin metabolism by amyloid-beta and presenilin. Nat. Cell. Biol..

[30] Zha Q., Ruan Y., Hartmann T., Beyreuther K., Zhang D. (2004). GM1 ganglioside regulates the proteolysis of amyloid precursor protein. Mol. Psychiatry.

[31] Grimm M.O., Zinser E.G., Grösgen S., Hundsdorfer B., Rothhaar T.L., Burg V.K., Kaestner L., Bayer T.A., Lipp P., Muller U. (2012). Amyloid precursor protein (APP) mediated regulation of ganglioside homeostasis linking Alzheimer’s disease pathology with ganglioside metabolism. PLoS One.

[32] Svennerholm L., Gottfries C.G. (1994). Membrane lipids, selectively diminished in Alzheimer brains, suggest synapse loss as a primary event in early-onset form (type I) and demyelination in late-onset form (type II). J. Neurochem.

[33] Wells K., Farooqui A.A., Liss L., Horrocks L.A. (1995). Neural membrane phospholipids in Alzheimer disease. Neurochem. Res.

[34] Prasad M.R., Lovell M.A., Yatin M., Dhillon H., Markesbery W.R. (1998). Regional membrane phospholipid alterations in Alzheimer’s disease. Neurochem. Res.

[35] Holmes O., Paturi S., Ye W., Wolfe M.S., Selkoe D.J. (2012). Effects of membrane lipids on the activity and processivity of purified gamma-secretase. Biochemistry.

[36] Simons K., Ikonen E. (1997). Functional rafts in cell membranes. Nature.

[37] Vetrivel K.S., Thinakaran G. (2010). Membrane rafts in Alzheimer’s disease beta-amyloid production. Biochim. Biophys. Acta.

[38] Riddell D.R., Christie G., Hussain I., Dingwall C. (2001). Compartmentalization of beta-secretase (Asp2) into low-buoyant density, noncaveolar lipid rafts. Curr. Biol.

[39] Vetrivel K.S., Cheng H., Lin W., Sakurai T., Li T., Nukina N., Wong P.C., Xu H., Thinakaran G. (2004). Association of gamma-secretase with lipid rafts in post-Golgi and endosome membranes. J. Biol. Chem.

[40] Vetrivel K.S., Cheng H., Kim S.H., Chen Y., Barnes N.Y., Parent A.T., Sisodia S.S., Thinakaran G. (2005). Spatial segregation of gamma-secretase and substrates in distinct membrane domains. J. Biol. Chem.

[41] Koumanov K.S., Tessier C., Momchilova A.B., Rainteau D., Wolf C., Quinn P.J. (2005). Comparative lipid analysis and structure of detergent-resistant membrane raft fractions isolated from human and ruminant erythrocytes. Arch. Biochem. Biophys.

[42] Kojro E., Gimpl G., Lammich S., Marz W., Fahrenholz F. (2001). Low cholesterol stimulates the nonamyloidogenic pathway by its effect on the α-secretase ADAM 10. Proc. Natl. Acad. Sci. USA.

[43] Ehehalt R., Keller P., Haass C., Thiele C., Simons K. (2003). Amyloidogenic processing of the Alzheimer beta-amyloid precursor protein depends on lipid rafts. J. Cell Biol.

[44] Parr-Sturgess C.A., Rushton D.J., Parkin E.T. (2010). Ectodomain shedding of the Notch ligand Jagged1 is mediated by ADAM17, but is not a lipid-raft-associated event. Biochem. J.

[45] Kuhn P.H., Wang H., Dislich B., Colombo A., Zeitschel U., Ellwart J.W., Kremmer E., Rossner S., Lichtenthaler S.F. (2010). ADAM10 is the physiologically relevant, constitutive alpha-secretase of the amyloid precursor protein in primary neurons. EMBO J.

[46] Stokes C.E., Hawthorne J.N. (1987). Reduced phosphoinositide concentrations in anterior temporal cortex of Alzheimer-diseased brains. J. Neurochem.

[47] Nitsch R.M., Blusztajn J.K., Pittas A.G., Slack B.E., Growdon J.H., Wurtman R.J. (1992). Evidence for a membrane defect in Alzheimer disease brain. Proc. Natl. Acad. Sci. USA.

[48] Nesic I., Guix F.X., Vennekens K., Michaki V., van Veldhoven P.P., Feiguin F., de Strooper B., Dotti C.G., Wahle T. (2012). Alterations in phosphatidylethanolamine levels affect the generation of Abeta. Aging Cell.

[49] Escriba P.V., Gonzalez-Ros J.M., Goni F.M., Kinnunen P.K., Vigh L., Sanchez-Magraner L., Fernandez A.M., Busquets X., Horvath I., Barcelo-Coblijn G. (2008). Membranes: A meeting point for lipids, proteins and therapies. J. Cell. Mol. Med.

[50] Bazan N.G., Scott B.L. (1990). Dietary omega-3 fatty acids and accumulation of docosahexaenoic acid in rod photoreceptor cells of the retina and at synapses. Ups. J. Med. Sci. Suppl.

[51] Ansari K.A., Shoeman D.W. (1990). Arachidonic and docosahexanoic acid content of bovine brain myelin: Implications for the pathogenesis of multiple sclerosis. Neurochem. Res.

[52] Horrocks L.A., Farooqui A.A. (2004). Docosahexaenoic acid in the diet: Its importance in maintenance and restoration of neural membrane function. Prostaglandins Leukot Essent Fatty Acids.

[53] Yang X., Sheng W., Sun G.Y., Lee J.C. (2011). Effects of fatty acid unsaturation numbers on membrane fluidity and alpha-secretase-dependent amyloid precursor protein processing. Neurochem. Int.

[54] Eckert G.P., Chang S., Eckmann J., Copanaki E., Hagl S., Hener U., Muller W.E., Kogel D. (2011). Liposome-incorporated DHA increases neuronal survival by enhancing non-amyloidogenic APP processing. Biochim. Biophys. Acta.

[55] Soderberg M., Edlund C., Kristensson K., Dallner G. (1991). Fatty acid composition of brain phospholipids in aging and in Alzheimer’s disease. Lipids.

[56] Tully A.M., Roche H.M., Doyle R., Fallon C., Bruce I., Lawlor B., Coakley D., Gibney M.J. (2003). Low serum cholesteryl ester-docosahexaenoic acid levels in Alzheimer’s disease: A case-control study. Br. J. Nutr.

[57] Cunnane S.C., Schneider J.A., Tangney C., Tremblay-Mercier J., Fortier M., Bennett D.A., Morris M.C. (2012). Plasma and brain fatty acid profiles in mild cognitive impairment and Alzheimer’s disease. J. Alzheimers Dis.

[58] Barberger-Gateau P., Letenneur L., Deschamps V., Peres K., Dartigues J.F., Renaud S. (2002). Fish, meat, and risk of dementia: Cohort study. Br. Med. J.

[59] Morris M.C., Evans D.A., Bienias J.L., Tangney C.C., Bennett D.A., Wilson R.S., Aggarwal N., Schneider J. (2003). Consumption of fish and n-3 fatty acids and risk of incident Alzheimer disease. Arch. Neurol.

[60] Schaefer E.J., Bongard V., Beiser A.S., Lamon-Fava S., Robins S.J., Au R., Tucker K.L., Kyle D.J., Wilson P.W., Wolf P.A. (2006). Plasma phosphatidylcholine docosahexaenoic acid content and risk of dementia and Alzheimer disease: The Framingham Heart Study. Arch. Neurol.

[61] Van Gelder B.M., Tijhuis M., Kalmijn S., Kromhout D. (2007). Fish consumption, n-3 fatty acids, and subsequent 5-y cognitive decline in elderly men: The Zutphen Elderly Study. Am. J. Clin. Nutr.

[62] Lukiw W.J., Cui J.G., Marcheselli V.L., Bodker M., Botkjaer A., Gotlinger K., Serhan C.N., Bazan N.G. (2005). A role for docosahexaenoic acid-derived neuroprotectin D1 in neural cell survival and Alzheimer disease. J. Clin. Invest.

[63] Perez S.E., Berg B.M., Moore K.A., He B., Counts S.E., Fritz J.J., Hu Y.S., Lazarov O., Lah J.J., Mufson E.J. (2010). DHA diet reduces AD pathology in young APPswe/PS1 Delta E9 transgenic mice: Possible gender effects. J. Neurosci. Res.

[64] Oksman M., Iivonen H., Hogyes E., Amtul Z., Penke B., Leenders I., Broersen L., Lutjohann D., Hartmann T., Tanila H. (2006). Impact of different saturated fatty acid, polyunsaturated fatty acid and cholesterol containing diets on beta-amyloid accumulation in APP/PS1 transgenic mice. Neurobiol. Dis.

[65] Calon F., Lim G.P., Yang F., Morihara T., Teter B., Ubeda O., Rostaing P., Triller A., Salem N., Ashe K.H. (2004). Docosahexaenoic acid protects from dendritic pathology in an Alzheimer’s disease mouse model. Neuron.

[66] Hooijmans C.R., Rutters F., Dederen P.J., Gambarota G., Veltien A., van Groen T., Broersen L.M., Lutjohann D., Heerschap A., Tanila H. (2007). Changes in cerebral blood volume and amyloid pathology in aged Alzheimer APP/PS1 mice on a docosahexaenoic acid (DHA) diet or cholesterol enriched Typical Western Diet (TWD). Neurobiol. Dis..

[67] Cole G.M., Ma Q.L., Frautschy S.A. (2009). Omega-3 fatty acids and dementia. Prostaglandins Leukot Essent Fatty Acids.

[68] Grimm M.O., Kuchenbecker J., Grösgen S., Burg V.K., Hundsdorfer B., Rothhaar T.L., Friess P., de Wilde M.C., Broersen L.M., Penke B. (2011). Docosahexaenoic acid reduces amyloid beta production via multiple pleiotropic mechanisms. J. Biol. Chem..

[69] Grziwa B., Grimm M.O., Masters C.L., Beyreuther K., Hartmann T., Lichtenthaler S.F. (2003). The transmembrane domain of the amyloid precursor protein in microsomal membranes is on both sides shorter than predicted. J. Biol. Chem.

[70] Duyckaerts C., Hauw J.J. (1997). Diagnosis and staging of Alzheimer disease. Neurobiol. Aging.

[71] Smith P.K., Krohn R.I., Hermanson G.T., Mallia A.K., Gartner F.H., Provenzano M.D., Fujimoto E.K., Goeke N.M., Olson B.J., Klenk D.C. (1985). Measurement of protein using bicinchoninic acid. Anal. Biochem.

[72] Grimm M.O., Grösgen S., Rothhaar T.L., Burg V.K., Hundsdorfer B., Haupenthal V.J., Friess P., Muller U., Fassbender K., Riemenschneider M. (2011). Intracellular APP domain regulates serine-palmitoyl-coa transferase expression and is affected in Alzheimer’s disease. Int. J. Alzheimers Dis..

[73] Bligh E.G., Dyer W.J. (1959). A rapid method of total lipid extraction and purification. Can. J. Biochem. Physiol.

[74] Grimm M.O., Kuchenbecker J., Rothhaar T.L., Grösgen S., Hundsdorfer B., Burg V.K., Friess P., Muller U., Grimm H.S., Riemenschneider M. (2011). Plasmalogen synthesis is regulated via alkyl-dihydroxyacetonephosphate-synthase by amyloid precursor protein processing and is affected in Alzheimer’s disease. J. Neurochem..

[75] Ruiz J.I., Ochoa B. (1997). Quantification in the subnanomolar range of phospholipids and neutral lipids by monodimensional thin-layer chromatography and image analysis. J. Lipid Res.

[76] Grimm M.O., Grösgen S., Riemenschneider M., Tanila H., Grimm H.S., Hartmann T. (2011). From brain to food: Analysis of phosphatidylcholins, lyso-phosphatidylcholins and phosphatidylcholin-plasmalogens derivates in Alzheimer’s disease human post mortem brains and mice model via mass spectrometry. J. Chromatogr. A.

