# Supplementary material for: Effect of Different Phospholipids on α-Secretase Activity in the Non-Amyloidogenic Pathway of Alzheimer’s Disease
Source: Int J Mol Sci. 2013 Mar 13;14(3):5879–98. doi: 10.3390/ijms14035879 (PMC3634454; doi:10.3390/ijms14035879)
Supplement: Supplementary file 1 [file ijms-14-05879-s001.pdf]

Supplementary Information

Figure S1. Effect of PC18:0 on α-secretase activity compared to the solvent control. Purified membranes of SH-SY5Y wt cells were incubated with 25 μM PC18:0 or the solvent EtOH. PC18:0 showed no alterations in α-secretase activity.

Figure S1: effect of PC 18:0 *vs* ethanol solvent control

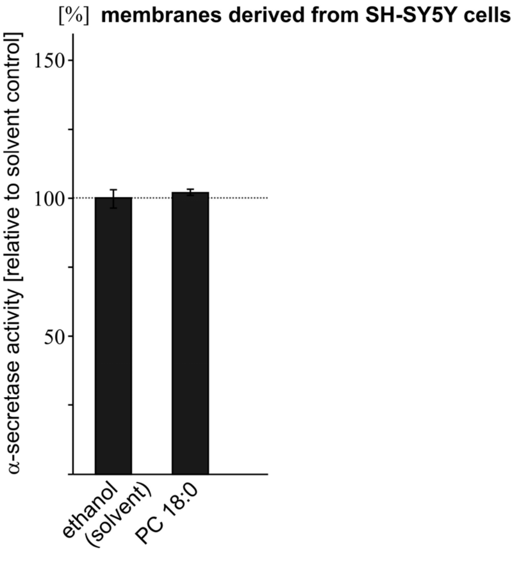

Figure S2. Ratio of phospholipid species in SH-SY5Y and human *post mortem* brain membranes. The ratio of the phospholipid species PC, PE and PS was determined by thin layer chromatography. The distribution of the three phospholipid species is represented below. PC is set to 100%. Both in SH-SY5Y cells and human brains PC is the major species followed by PE and PS. However, the distribution between PC:PE:PS is slightly different between the neuroblastoma cell lines and the membrane derived from human brains.

Figure S2: distribution of headgroups in membranes derived from SH-SY5Y and human brains

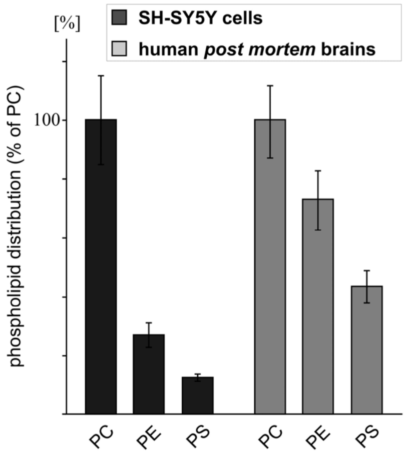

Figure S3. Representative kinetics for α-secretase measurements. (**A**) PC18:0 *versus* solvent control ethanol. Determination of α-secretase activity in the presence of PC18:0 (25 µM) in purified membranes of SH-SY5Y cells. The α-secretase activity was determined by a fluorometric assay; (**B**) PC10:0–PC18:0 *in vitro*. Determination of α-secretase activity in the presence of PC10:0–PC18:0: Influence of the fatty acid chain length on α-secretase activity in purified membranes of human SH-SY5Y cells. Purified membranes of SH-SY5Y cells were incubated with the phospholipids (25 µM), PC18:0 served as control. The α-secretase activity was determined by a fluorometric assay. α-secretase activity was increased by PC10:0, PC12:0 and PC14:0 and not by PC16:0. Error bars represent SEM (Standard error of the mean); (**C**) PC18:0, PC20:4, PC20:5, PC22:6 *in vitro*. Determination of α-secretase activity in the presence of PC18:0–PC22:6: Influence of the saturation grade on α-secretase activity in purified membranes of human SH-SY5Y cells. Purified membranes of SH-SY5Y cells were incubated with the phospholipids (25 µM), PC18:0 served as control. α-secretase activity was determined by a fluorometric assay. The α-secretase activity was increased by PC20:4 and PC22:6. The strongest effect can be seen for PC20:5. Error bars represent SEM (Standard error of the mean); (**D**) PC12:0–PC18:0 *in vivo*. Determination of α-secretase activity in the presence of PC10:0–PC18:0: Influence of the fatty acid chain length on α-secretase activity in human SH-SY5Y cells. SH-SY5Y cells were incubated with the phospholipids (10 µM) for 8 + 16 h. α-secretase activity was determined by a fluorometric live cell assay. The α-secretase activity was increased by PC10:0, PC12:0 and PC14:0 and not by PC16:0. Error bars represent SEM (Standard error of the mean); (**E**) PC18:0, PC 20:4, PC20:5, PC22:6 *in vivo*. Determination of α-secretase activity in the presence of PC18:0–PC22:6: Influence of the saturation grade on α-secretase activity in human SH-SY5Y cells. SH-SY5Y cells were incubated with the phospholipids (10 µM) for 8 + 16 h. The α-secretase activity was determined by a fluorometric live cell assay. The α-secretase activity was increased by all unsaturated PC species, PC18:2 and PC18:3 showed minor effects, the strongest effect can be seen for PC20:5 and PC22:6. Error bars represent SEM (Standard error of the mean); (**F**) Linear regression of the kinetics shown in E; (**G**) PC12:0, PC 18:0, purified ADAM10. Determination of ADAM10 activity in the presence of PC12:0 and PC18:0 (control): ADAM10 purified enzyme was treated with the phospholipids (25 µM) in the presence of a human brain lipid environment. ADAM10 activity was determined by a fluorometric assay. PC12:0 showed a significant increase of activity compared to PC18:0. Error bars represent SEM (Standard error of the mean); (**H**) PC18:0, PC22:6, purified ADAM10. Determination of ADAM10 activity in the presence of unsaturated phospholipids PC18:3 and PC22:6, PC18:0 served as a control: ADAM10 purified enzyme was treated with the phospholipids (25 µM) in the presence of a human brain lipid environment. ADAM10 activity was determined by a fluorometric assay. PC18:3 and PC22:6 showed significantly increased activity of ADAM10 compared to PC18:0. Error bars represent SEM (Standard error of the mean); (**I**) PC12:0, PC18:0, human *post mortem* brain. Influence on α-secretase activity *ex vivo* in purified membranes of human *post mortem* brains. Purified brain membranes were incubated with PC12:0 and

the corresponding control PC18:0 (50 μM). Afterwards, α-secretase activity was measured by a fluorometric assay. PC12:0 increased α-secretase activity significantly compared to PC18:0. Error bars represent SEM (Standard error of the mean); (**J**) PC18:0, PC22:6, human *post mortem* brains. Influence on α-secretase activity *ex vivo* in purified membranes of human *post mortem* brains. Purified brain membranes were incubated with unsaturated phospholipids PC18:3 and PC22:6 and the corresponding control PC18:0 (50 μM). Afterwards, α-secretase activity was measured by a fluorometric assay. PC18:3 showed a moderate increase in the activity of α-secretase, whereas PC22:6 significantly enhanced α-secretase activity compared to PC18:0. Error bars represent SEM (Standard error of the mean).

Figure S3: representative kinetics for α-secretase measurements

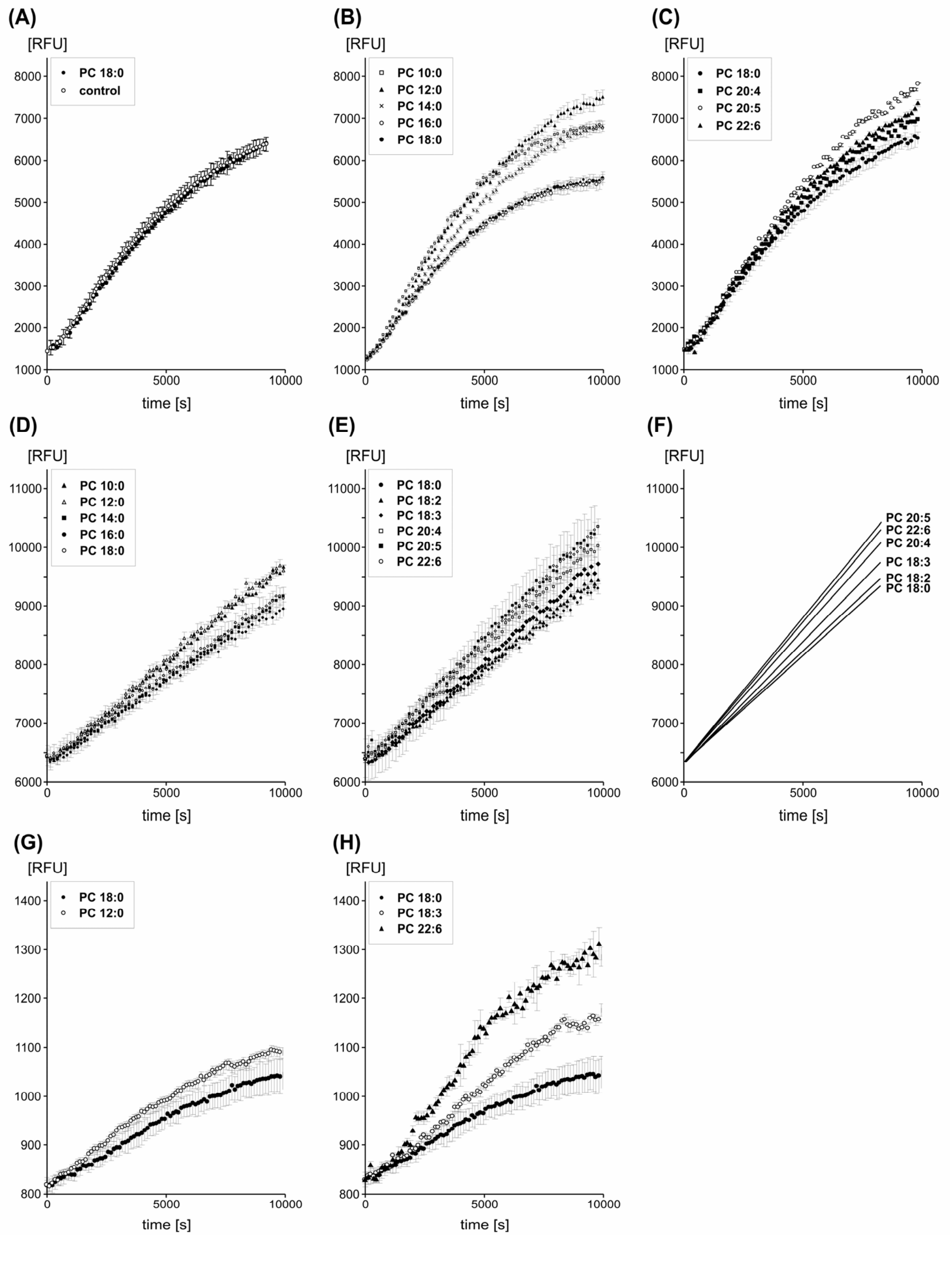

Figure S3. *Cont.*

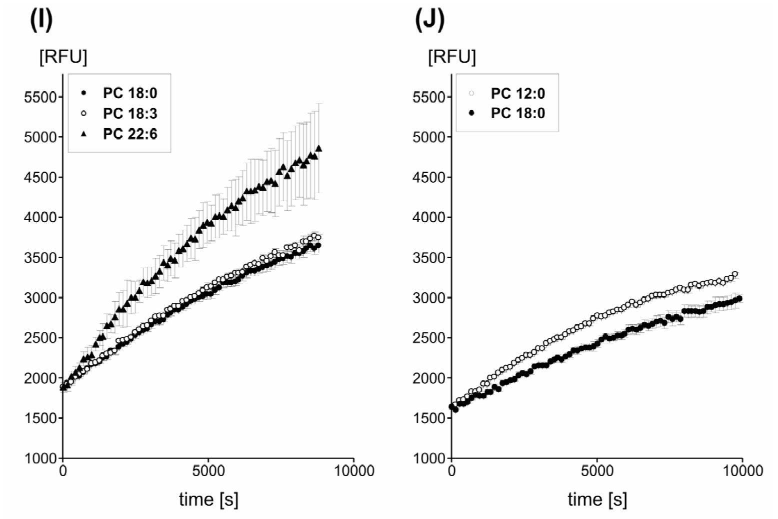

Figure S4. Specificity of α-secretase activity assay. (**A**) Specificity of α-secretase activity measurement was analyzed by adding the α-secretase inhibitors GM6001 (100 µM), Phenanthroline (1 mM) and EDTA/EGTA (1 mM) to purified membranes of SH-SY5Y cells; (**B**) For validation of the α-secretase measurement on living SH-SY5Y cells, the sAPPα level was determined by the use of the antibody W02 as described in Ida *et al*., 1996.

Figure S4: Specificity of α-secretase activity assay

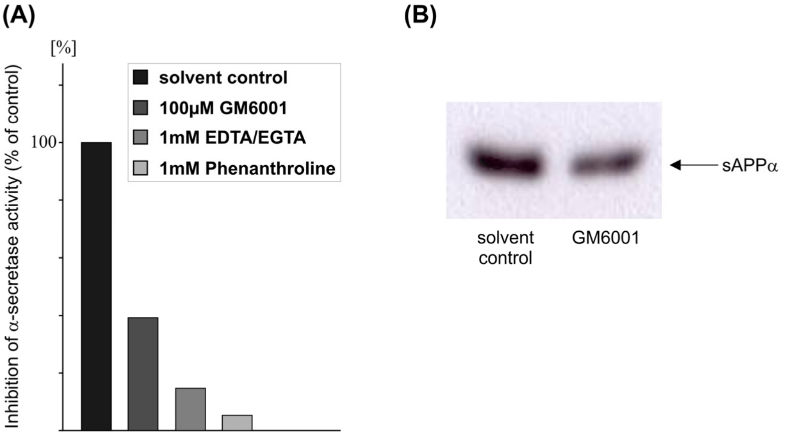

Figure S5. Phospholipid uptake into SH-SY5Y cells. SH-SY5Y cells were incubated with phospholipids (**A**) PC12:0 and (**B**) PC 18:0. Phospholipid uptake into SH-SY5Y cells was measured via mass spectrometry. Arrows represent specific signal for the incubated phospholipid. Additionally, the phospholipid incorporation into SH-SY5Y membranes was measured. cps (counts per second) for PC12:0 is 116,682.4 +/− 10,977.5, corresponding control is 133.8 +/− 9.9. PC18:0 is 116,111.3 +/− 8892.1 cps, the corresponding control is 2371.9 +/− 1668 cps.

Figure S5: Phospholipid uptake into SH-SY5Y cells

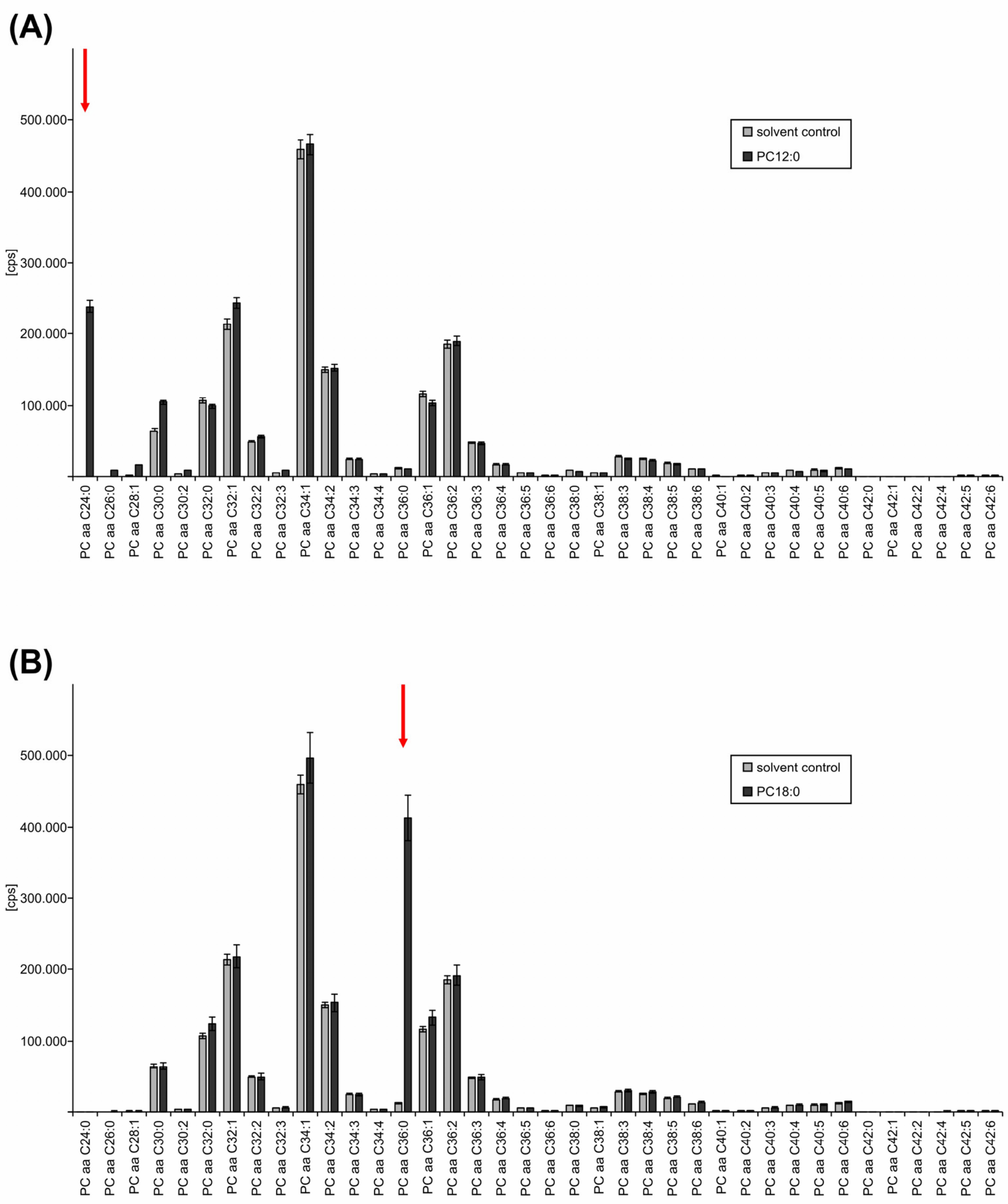

Statistical Analysis of Phospholipid Species on α-Secretase Activity.

Statistical analysis was performed by ANOVA test for SH-SY5Y and human brain lipid environment. F values from analysis of variance and the corresponding p-values are shown. Two-tailed Student's *t*-test was performed for analysis of FA chain length in human brain lipid environment. *p*-values determined by *post hoc* test are listed below. Additionally, mean values and SEM for each phospholipid are displayed. Effect of phospholipid species were analyzed for each method alone (first two lanes) and, additionally, in a combined data set for SH-SY5Y membranes and living cells (last lanes) and a combined data set for human brain membranes and ADAM10 in human brain lipid environment. (**S1**) **Effect of FA chain length** Effect of chain length was analyzed for PC 10:0 to PC 24:0 in SH-SY5Y cells, and for PC18:0 and PC12:0 in human brain lipid environment. Statistical analysis of human brain lipid environment was performed here by Student's *t*-test. (**S2**) **Effect of phospholipid headgroup** The effect of the headgroup on -secretase activity was analyzed for PC / PE / PS 12:0 and 14:0 on SH-SY5Y cells and human brain lipid environment. (**S3**) **Effect of FA saturation** The effect of phospholipid saturation on -secretase activity was determined for PC18:0 to PC18:3, PC20:4, PC20:5 and PC22:6 in SH-SY5Y cells and PC18:0, PC18:3, PC22:6 in human brain lipid environment. (**S4**) **Effect of double-bond position** The effect of phospholipid double-bond position on -secretase activity was determined for PC18:1Δ9-cis and PC18:1Δ6 in SH-SY5Y cells.

Table S1. Effect of chain length.

SH-SY5Y	membranes (a)	ANOVA: $F(7,16) = 8.77$, $p < 0.001$
	living cells (b)	ANOVA: $F(7,76) = 32.00$, $p < 0.001$
	combined data (a+b)	ANOVA: $F(7,92) = 33.13$, $p < 0.001$
human brain lipid environment	membranes (c)	ttest 2 tailed 12:0 vs 18:0 $p = 0.004$
	purified ADAM 10 (d)	ttest 2 tailed 12:0 vs 18:0 $p = 0.011$
	combined data (c+d)	ttest 2 tailed 12:0 vs 18:0 $p < 0.001$

Mean & SEM%:

	SH-SY5Y membranes (a)		living SH-SY5Y cells (b)		combined data (a+b)	
	Mean	SEM%	Mean	SEM%	Mean	SEM%
PC 10:0	127.71	2.23	124.75	1.30	126.02	1.25
PC 12:0	143.98	9.31	126.91	1.28	129.92	2.38
PC 14:0	127.99	11.92	103.52	1.24	114.01	6.72
PC 16:0	99.81	5.91	99.20	1.29	99.30	1.33
PC 18:0	100.00	3.20	100.00	2.29	100.00	1.98
PC 20:0	102.32	0.53	98.81	1.89	99.68	1.48
PC 22:0	98.77	1.90	100.08	2.26	99.77	1.76
PC 24:0	101.67	1.52	107.82	2.18	106.40	1.84

Table S1. *Cont.*

	human brain lipid environment					
	**membranes (c)**		**purified ADAM10 (d)**		**combined data (c+d)**	
	Mean	SEM%	Mean	SEM%	Mean	SEM%
18:0	100.00	5.69	100.00	6.40	100.00	4.99
12:0	141.86	7.55	132.01	9.60	134.97	7.05

Post-Hoc Test (p-values)

		SH-SY5Y membranes (a)	living SH-SY5Y cells (b)	combined data (a+b)
PC 10:0	PC 12.0	0.8761	1.0000	0.9996
	PC 14.0	1.0000	**0.0005**	0.0816
	PC 16.0	0.1178	**0.0000**	**0.0000**
	PC 18.0	0.1233	**0.0000**	**0.0000**
	PC 20.0	0.2090	**0.0000**	**0.0000**
	PC 22.0	0.0920	**0.0000**	**0.0000**
	PC 24.0	0.1808	**0.0012**	**0.0000**
PC 12:0	PC 10.0	0.8761	1.0000	0.9996
	PC 14.0	0.8918	**0.0000**	**0.0002**
	PC 16.0	**0.0023**	**0.0000**	**0.0000**
	PC 18.0	**0.0024**	**0.0000**	**0.0000**
	PC 20.0	**0.0042**	**0.0000**	**0.0000**
	PC 22.0	**0.0018**	**0.0000**	**0.0000**
	PC 24.0	**0.0036**	**0.0000**	**0.0000**
PC 14:0	PC 10.0	1.0000	**0.0005**	0.0816
	PC 12.0	0.8918	**0.0000**	**0.0002**
	PC 16.0	0.1104	0.9996	**0.0005**
	PC 18.0	0.1155	1.0000	**0.0011**
	PC 20.0	0.1966	0.9995	**0.0027**
	PC 22.0	0.0861	1.0000	**0.0024**
	PC 24.0	0.1698	0.9999	0.5795
PC 16:0	PC 10.0	0.1178	**0.0000**	**0.0000**
	PC 12.0	**0.0023**	**0.0000**	**0.0000**
	PC 14.0	0.1104	0.9996	**0.0005**
	PC 18.0	1.0000	1.0000	1.0000
	PC 20.0	1.0000	1.0000	1.0000
	PC 22.0	1.0000	1.0000	1.0000
	PC 24.0	1.0000	**0.0479**	0.2204
PC 18:0	PC 10.0	0.1233	**0.0000**	**0.0000**
	PC 12.0	**0.0024**	**0.0000**	**0.0000**
	PC 14.0	0.1155	1.0000	**0.0011**
	PC 16.0	1.0000	1.0000	1.0000
	PC 20.0	1.0000	1.0000	1.0000
	PC 22.0	1.0000	1.0000	1.0000
	PC 24.0	1.0000	0.1032	0.3777
PC 20:0	PC 10.0	0.2090	**0.0000**	**0.0000**
	PC 12.0	**0.0042**	**0.0000**	**0.0000**
	PC 14.0	0.1966	0.9995	**0.0027**
	PC 16.0	1.0000	1.0000	1.0000
	PC 18.0	1.0000	1.0000	1.0000
	PC 22.0	1.0000	1.0000	1.0000
	PC 24.0	1.0000	0.1038	0.5126

Table S1. *Cont.*

		SH-SY5Y membranes (a)	living SH-SY5Y cells (b)	combined data (a+b)
PC 22:0	PC 10.0	0.0920	**0.0000**	**0.0000**
	PC 12.0	**0.0018**	**0.0000**	**0.0000**
	PC 14.0	0.0861	1.0000	**0.0024**
	PC 16.0	1.0000	1.0000	1.0000
	PC 18.0	1.0000	1.0000	1.0000
	PC 20.0	1.0000	1.0000	1.0000
	PC 24.0	1.0000	0.2539	0.4985
PC 24:0	PC 10.0	0.1808	**0.0012**	**0.0000**
	PC 12.0	**0.0036**	**0.0000**	**0.0000**
	PC 14.0	0.1698	0.9999	0.5795
	PC 16.0	1.0000	**0.0479**	0.2204
	PC 18.0	1.0000	0.1032	0.3777
	PC 20.0	1.0000	0.1038	0.5126
	PC 22.0	1.0000	0.2539	0.4985

Table S2. Effect of head group.

SH-SY5Y	membranes	FA 12:0 (a1)	ANOVA: $F(2,11) = 3{,}29$, $p = 0.076$
		FA 14:0 (a2)	ANOVA: $F(2,11) = 15{,}93$, $p < 0.001$
		FA combined (a1&a2)	ANOVA: $F(2,25) < 1{,}0$, $p = 0.787$
	living cells	FA 12:0 (b1)	ANOVA: $F(2,6) = 17.52$, $p = 0.003$
		FA 14:0 (b2)	ANOVA: $F(2,6) = 2.49$, $p = 0.163$
		FA combined (b1&b2)	ANOVA: $F(2,15) = 4{,}25$, $p = 0.035$
	combined data membranes and living cells (a&b)		ANOVA: $F(2,43) < 1{,}0$, $p = 0.502$
human brain membranes	membranes	FA 12:0 (c1)	ANOVA: $F(2,14) = 1{,}37$, $p = 0.286$
		FA 14:0 (c2)	ANOVA: $F(2,6) < 1{,}0$, $p = 0.908$
		FA combined (c1&c2)	ANOVA: $F(2,23) = 1{,}08$, $p = 0.358$

Mean & SEM%

	SH-SY5Y					
	membranes					
	FA 12:0 (a1)		FA 14:0 (a2)		FA combined (a1&a2)	
	Mean	SEM%	Mean	SEM%	Mean	SEM%
PC	100.00	0.66	100.00	1.89	100.00	1.15
PE	98.48	1.28	100.95	1.54	99.71	1.02
PS	93.42	2.47	116.18	3.12	101.96	4.53

	SH-SY5Y						combined living cells and membranes (a+b)	
	living cells							
	FA 12:0 (b1)		FA 14:0 (b2)		FA combined (b1&b2)			
	Mean	SEM%	Mean	SEM%	Mean	SEM%	Mean	SEM%
PC	100.00	1.10	100.00	1.08	100.00	0.69	100.00	0.70
PE	93.63	2.75	104.99	4.04	99.31	3.35	99.58	1.25
PS	83.81	1.62	96.57	2.04	90.19	3.08	96.91	3.24

Table S2. *Cont.*

	human brain membranes					
	FA 12:0 (c1)		FA 14:0 (c2)		FA combined (c1&c2)	
	Mean	SEM%	Mean	SEM%	Mean	SEM%
PC	100.00	4.50	100.00	10.90	100.00	4.28
PE	109.24	3.05	105.68	3.55	107.91	2.26
PS	104.85	3.68	104.67	12.20	104.79	4.25

Post-Hoc Test (p-values)

		SH-SY5Y						
		membranes			living cells			combined data membranes and living cells (a&b)
		FA 12:0 (a1)	FA 14:0 (a2)	FA combined (a1&a2)	FA 12:0 (b1)	FA 14:0 (b2)	FA combined (b1&b2)	
PC	PE	0.935	0.978	1.000	0.170	0.555	0.997	0.998
	PS	0.128	**0.001**	0.936	**0.003**	0.785	0.058	0.639
PE	PC	0.935	0.978	1.000	0.170	0.555	0.997	0.998
	PS	0.169	**0.001**	0.883	**0.035**	0.191	0.082	0.698
PS	PC	0.128	**0.001**	0.936	**0.003**	0.785	0.058	0.639
	PE	0.169	**0.001**	0.883	**0.035**	0.191	0.082	0.698

		human brain membranes		
		FA 12:0 (c1)	FA 14:0 (c2)	FA combined (c1&c2)
PC	PE	0.322	0.971	0.410
	PS	0.761	0.983	0.756
PE	PC	0.322	0.971	0.410
	PS	0.831	1.000	0.923
PS	PC	0.761	0.983	0.756
	PE	0.831	1.000	0.923

Table S3. Effect of saturation.

SH-SY5Y	membranes (a)	ANOVA: F(6,23) = 4,95, p = 0.002
	living cells (b)	ANOVA: F(6,56) = 11,39, p < 0.001
	combined data (a+b)	ANOVA: F(6,79) = 9,77, p < 0.001
human brain lipid enviroment	membranes (c)	ANOVA: F(2,12) = 7,80, p = 0.007
	purified ADAM 10 (d)	ANOVA: F(2,9) = 182,87, p < 0.001
	combined data (c+d)	ANOVA: F(2,24) = 18,41, p < 0.001

Mean & SEM%

	SH-SY5Y					
	membranes (a)		living cells (b)		combined data (a+b)	
	Mean	SEM%	Mean	SEM%	Mean	SEM%
18:0	100.00	1.17	100.00	2.29	100.00	1.77
18:1	101.96	1.54	110.32	4.33	108.04	3.33
18:2	97.11	2.10	115.00	6.60	110.12	5.36
18:3	98.18	1.99	117.40	2.31	112.15	3.20
20:4	104.21	4.78	124.70	1.97	117.87	3.49
20:5	123.08	7.63	140.05	14.18	132.51	8.23
22:6	108.56	2.63	136.82	2.22	123.52	3.89

	human brain lipid environment					
	membranes (c)		purified ADAM10 (d)		combined data (c+d)	
	Mean	SEM%	Mean	SEM%	Mean	SEM%
18:0	100.00	3.08	100.00	4.76	100.00	2.63
18:3	106.28	2.65	148.17	4.21	127.23	9.63
22:6	151.98	17.40	229.70	2.72	181.13	17.64

Post-Hoc Test (p-values)

		SH-SY5Y		
		membranes (a)	living cells (b)	combined data (a+b)
18:0	18:1	1.000	0.802	0.791
	18:2	1.000	0.201	0.404
	18:3	1.000	0.068	0.142
	20:4	1.000	**0.001**	**0.002**
	20:5	**0.005**	**0.000**	**0.000**
	22:6	0.791	**0.000**	**0.000**
18:1	18:0	1.000	0.802	0.791
	18:2	1.000	1.000	1.000
	18:3	1.000	0.999	1.000
	20:4	1.000	0.516	0.674
	20:5	**0.042**	**0.005**	**0.000**
	22:6	0.996	**0.003**	**0.026**
18:2	18:0	1.000	0.201	0.404
	18:1	1.000	1.000	1.000
	18:3	1.000	1.000	1.000
	20:4	0.998	0.965	0.938
	20:5	**0.006**	**0.033**	**0.002**
	22:6	0.615	**0.026**	0.096

Table S3. *Cont.*

		SH-SY5Y		
		membranes (a)	living cells (b)	combined data (a+b)
18:3	18:0	1.000	0.068	0.142
	18:1	1.000	0.999	1.000
	18:2	1.000	1.000	1.000
	20:4	1.000	0.999	0.998
	20:5	**0.009**	0.080	**0.006**
	22:6	0.763	0.077	0.288
20:4	18:0	1.000	**0.001**	**0.002**
	18:1	1.000	0.516	0.674
	18:2	0.998	0.965	0.938
	18:3	1.000	0.999	0.998
	20:5	0.056	0.633	0.133
	22:6	1.000	0.754	0.993
20:5	18:0	**0.005**	**0.000**	**0.000**
	18:1	**0.042**	**0.005**	**0.000**
	18:2	**0.006**	**0.033**	**0.002**
	18:3	**0.009**	0.080	**0.006**
	20:4	0.056	0.633	0.133
	22:6	0.131	1.000	0.790
22:6	18:0	0.791	**0.000**	**0.000**
	18:1	0.996	**0.003**	**0.026**
	18:2	0.615	**0.026**	0.096
	18:3	0.763	0.077	0.288
	20:4	1.000	0.754	0.993
	20:5	0.131	1.000	0.790

		human brain lipid enviroment		
		membranes (c)	purified ADAM 10 (d)	combined data (c+d)
18:0	18:3	0.974	**0.000**	0.212
	22:6	**0.007**	**0.000**	**0.000**
18:3	18:0	0.974	**0.000**	0.212
	22:6	0.058	**0.000**	**0.008**
22:6	18:0	**0.007**	**0.000**	**0.000**
	18:3	0.058	**0.000**	**0.008**

Table S4. Effect of double bond position.

SH-SY5Y	membranes	ttest 2 tailed 18:1D9 (cis) vs 18:1D6, p = 0.041
	living cells	ttest 2 tailed 18:1D9 (cis) vs 18:1D6, p = 0.78

Mean & SEM%:

	SH-SY5Y			
	membranes		living cells	
	Mean	SEM%	Mean	SEM%
18:1D9 (cis)	100.00	1.76	100.00	4.40
18:1D6	106.08	0.96	101.65	4.45

Table S5. Lipid composition of membranes derived from human *post mortem* brain and SH-SY5Y cell membranes. The metabolites of the fatty acids are measured from lipid extracts of human brain and SH-SY5Y membranes. The metabolites are listed in the first lane, the Q1 and Q2 masses of the metabolites were detected by mass spectrometry. PC aa and PC ae represent both phospholipids, PC ae are plasmalogens. PC C 34:1 is set to 100%. The other phospholipid species are given in % to PC C 34:1.

PC Lipid composition of membranes derived from SH-SY5Y and human post mortem brains
Samples were normalized to PC aa 34:1 and PCae 34:1

**Metabolite**	**Q1 Mass (Da)**	**Q3 Mass (Da)**	**SH-SY5Y Mean%**	**human brain Mean%**	**SH-SY5Y SEM%**	**human brain SEM%**
PC aa C24:0	622.4	184	0.330	0.062	0.028	0.006
PC aa C26:0	650.5	184	1.167	0.609	0.095	0.065
PC aa C28:1	676.5	184	0.689	0.161	0.027	0.013
PC aa C30:0	706.5	184	12.592	3.486	0.063	0.025
PC aa C30:2	702.5	184	0.724	0.083	0.009	0.004
PC aa C32:0	734.6	184	24.145	32.487	0.066	0.103
PC aa C32:1	732.6	184	37.181	19.681	0.100	0.122
PC aa C32:2	730.5	184	8.300	1.437	0.025	0.010
PC aa C32:3	728.5	184	0.886	0.042	0.004	0.000
PC aa C34:1	760.6	184	100.000	100.000		
PC aa C34:2	758.6	184	26.674	5.550	0.078	0.023
PC aa C34:3	756.6	184	3.762	0.324	0.012	0.004
PC aa C34:4	754.5	184	0.411	0.098	0.008	0.002
PC aa C36:0	790.6	184	2.635	2.896	0.010	0.007
PC aa C36:1	788.6	184	27.707	29.832	0.057	0.107
PC aa C36:2	786.6	184	36.188	13.058	0.138	0.045
PC aa C36:3	784.6	184	8.385	3.885	0.041	0.018
PC aa C36:4	782.6	184	2.340	8.794	0.010	0.022
PC aa C36:5	780.6	184	0.803	0.329	0.066	0.004
PC aa C36:6	778.5	184	0.264	0.103	0.017	0.003
PC aa C38:0	818.7	184	1.311	0.349	0.005	0.003
PC aa C38:1	816.7	184	1.308	3.031	0.005	0.027
PC aa C38:3	812.6	184	5.881	2.817	0.016	0.021
PC aa C38:4	810.6	184	4.328	9.055	0.023	0.037
PC aa C38:5	808.6	184	2.940	3.979	0.149	0.050
PC aa C38:6	806.6	184	1.575	5.285	0.042	0.026
PC aa C40:1	844.7	184	0.285	0.387	0.005	0.003
PC aa C40:2	842.7	184	0.475	1.446	0.001	0.012

Table S5. *Cont.*

Metabolite	Q1 Mass (Da)	Q3 Mass (Da)	SH-SY5Y Mean%	human brain Mean%	SH-SY5Y SEM%	human brain SEM%
PC aa C40:3	840.7	184	1.333	0.365	0.006	0.003
PC aa C40:4	838.6	184	1.845	1.120	0.010	0.006
PC aa C40:5	836.6	184	1.562	1.235	0.038	0.017
PC aa C40:6	834.6	184	1.772	3.195	0.054	0.013
PC aa C42:0	874.7	184	0.172	0.088	0.009	0.001
PC aa C42:1	872.7	184	0.143	0.339	0.003	0.003
PC aa C42:2	870.7	184	0.104	0.388	0.002	0.005
PC aa C42:4	866.7	184	0.192	0.125	0.002	0.001
PC aa C42:5	864.7	184	0.214	0.094	0.004	0.002
PC aa C42:6	862.6	184	0.334	0.182	0.007	0.010
PC ae C30:0	692.6	184	5.950	2.271	0.055	0.038
PC ae C30:1	690.5	184	1.780	1.351	0.043	0.085
PC ae C30:2	688.5	184	0.298	0.404	0.023	0.036
PC ae C32:1	718.6	184	25.050	10.680	0.100	0.089
PC ae C32:2	716.6	184	4.337	0.979	0.013	0.034
PC ae C34:0	748.6	184	22.981	32.355	0.169	0.209
PC ae C34:1	746.6	184	100.000	100.000		
PC ae C34:2	744.6	184	21.120	5.621	0.121	0.084
PC ae C34:3	742.6	184	3.339	0.835	0.021	0.038
PC ae C36:0	776.7	184	3.969	8.509	0.085	0.066
PC ae C36:1	774.6	184	22.747	50.795	0.090	0.210
PC ae C36:2	772.6	184	30.646	21.567	0.145	0.111
PC ae C36:3	770.6	184	16.933	3.142	0.127	0.046
PC ae C36:4	768.6	184	6.152	5.826	0.015	0.047
PC ae C36:5	766.6	184	1.213	1.045	0.047	0.020
PC ae C38:0	804.7	184	1.953	5.212	0.032	0.046
PC ae C38:1	802.7	184	4.478	18.418	0.039	0.221
PC ae C38:2	800.7	184	8.199	13.103	0.043	0.067
PC ae C38:3	798.6	184	14.828	3.914	0.050	0.051
PC ae C38:4	796.6	184	12.293	8.464	0.066	0.097
PC ae C38:5	794.6	184	10.873	5.829	0.116	0.034
PC ae C38:6	792.6	184	6.059	3.867	0.038	0.044
PC ae C40:0	832.7	184	5.060	24.187	0.065	0.126
PC ae C40:1	830.7	184	3.664	19.827	0.269	0.397
PC ae C40:2	828.7	184	1.781	35.134	0.016	0.364
PC ae C40:3	826.7	184	4.683	3.419	0.026	0.040
PC ae C40:4	824.7	184	7.047	3.920	0.033	0.046
PC ae C40:5	822.6	184	5.952	3.069	0.033	0.037
PC ae C40:6	820.6	184	6.329	3.586	0.050	0.062
PC ae C42:0	860.8	184	1.019	3.461	0.035	0.052
PC ae C42:1	858.7	184	0.732	3.417	0.021	0.065
PC ae C42:2	856.7	184	0.616	3.967	0.020	0.097
PC ae C42:3	854.7	184	0.749	3.121	0.027	0.045
PC ae C42:4	852.7	184	0.812	0.701	0.007	0.014
PC ae C42:5	850.7	184	0.848	0.829	0.007	0.032
PC ae C44:3	882.7	184	0.416	1.058	0.039	0.021
PC ae C44:4	880.7	184	0.259	0.876	0.009	0.037
PC ae C44:5	878.7	184	0.244	1.692	0.008	0.033
PC ae C44:6	876.7	184	0.337	0.806	0.006	0.036

© 2013 by the authors; licensee MDPI, Basel, Switzerland. This article is an open access article distributed under the terms and conditions of the Creative Commons Attribution license (http://creativecommons.org/licenses/by/3.0/).
